# Histone deacetylase inhibitor induced pVHL-independent degradation of HIF-1α and hierarchical quality control of pVHL via chaperone system

**DOI:** 10.1371/journal.pone.0248019

**Published:** 2021-07-30

**Authors:** Jieming Ni, Anping Ni

**Affiliations:** Chinese Academy of Medical Sciences and Peking Union Medical College, Beijing, P.R. China; Technische Universitat Munchen, TranslaTUM, GERMANY

## Abstract

The mortality rate of ovarian cancer is increasing and the role of hypoxia inducible factor-1α (HIF-1α) in tumor progression has been confirmed. von Hippel-Lindau tumor suppressor protein (pVHL) binds HIF-1α and mediates proteasome degradation of HIF-1α. Besides, histone deacetylase inhibitor (HDACi) mitigates tumor growth via targeting HIF-1α, whereas underlying mechanism still requires investigation. In this research, we exposed ovarian cancer cell lines OV-90 and SKOV-3 to escalating concentrations of HDACi LBH589. As a result, cell viability was significantly suppressed and expression of HIF-1α was remarkably reduced along with decreased levels of signal molecules, including phosphoinositide 3-kinase (PI3K) and glycogen synthase kinase 3β (GSK3β) (P = 0.000). Interestingly, pVHL was expressed in a notably declining tendency (P = 0.000). Chaperone heat shock protein-70 (HSP70) was expressed in an ascending manner, whereas expression of chaperonin TCP-1α was reduced clearly (P = 0.000). Besides, co-inhibition of pVHL plus HDAC did not contribute to a remarkable difference in HIF-1α expression as compared with single HDAC inhibition. Furthermore, both cell lines were transfected with plasmids of VHL plus VHL binding protein-1 (VBP-1). Consequently, the expression of HIF-1α as well as lactate dehydrogenase-A (LDHA) was remarkably decreased (P = 0.000). These findings indicate HDACi may repress expression of HIF-1α via inhibiting PI3K and GSK3β and promote degradation of HIF-1α via HSP70, independent of pVHL. Additionally, a sophisticated network of HDAC and chaperones may involve in pVHL quality control.

## Introduction

Ovarian cancer has been the eighth most common disease leading to death and the five-year survival rate of patients with ovarian cancer is lower than 45% around the world [1, 2]. Resistances and relapses are great challenges to the treatment of advanced stage patients, who are prone to develop refractory diseases after cytoreductive surgeries and chemotherapies [3].

Hypoxia inducible factor-1α (HIF-1α) is cellular expression product for hypoxic response due to hyperactive proliferation of tumor, and many studies have proved the correlation between HIF-1α and metastasis, angiogenesis and resistance [4, 5]. It has been clear that von Hippel-Lindau tumor suppressor protein (pVHL) plays a major role in HIF-1α degradation. The stability and function of pVHL rely on a sophisticated chaperone machine [6]. Under normoxic conditions, HIF-1α with both hydroxylation and acetylation is ready for recognition by pVHL and further undergoes ubiquitin-proteasome degradation, whereas hypoxia interferes this post-translational modification [5]. HIF-1α expression was found with a significantly increased activity on the margin of tumor infiltration and necrosis surroundings and its accumulation in hypoxia further prompts epithelial-mesenchymal transition [7–9]. As a crucial metabolic reprogramming factor, HIF-1α may be involved in the progression of a borderline ovarian tumor to an epithelial ovarian cancer, which entails hypoxia-induced invasiveness and migration [10]. Daponte et al. [11] reported that HIF-1α expression in benign ovarian lesions was significantly lower than that in borderline tumors and malignant tumors (P < 0.001). And the expression of HIF-1α was also found to be associated with malignant degree, FIGO stage, lymph node metastasis and overall survival of patients [12, 13]. Moreover, previous literature has proved hypoxia induces the downregulation of influx transporters including organic cationic transporter, which is responsible for chemotherapy regimens uptake and contributes to the acquired chemo-resistance [14, 15]. Besides, hypoxia response element (HRE) of HIF-1α was also found on multidrug resistance gene-1 (MDR1) promoter region and HIF-1α binding to HRE not only gives rise to vigorous cellular metabolism but also induces upregulation of MDR1/P-gp, consequently increasing resistance [16, 17]. Finally, activation of phosphoglycerate dehydrogenase in the process of metabolic reprogramming contributes to an increased cellular redox potential, which ultimately mitigates efficacy of cytotoxic regimens and enhances resistance [18].

Histone deacetylase (HDAC) and histone acetyltransferase (HAT) modulate transcription machinery via reversible acetylation, and the role of HDAC in regulating stability and activity of HIF-1α has been depicted yet [19]. JNK-sustaining HDAC6 expression may promote HIF-1α stability indirectly through the deacetylation of heat shock protein 90 (HSP90), which facilitates chaperone function of HSP90 toward HIF-1α [20]. Panobinostat (LBH589), a novel pan-HDAC inhibitor (HDACi), is confirmed with a potent inhibitory capability in suppressing HIF-1α. The research of Yao et al. [21] showed that LBH589 might inhibit the deacetylase activity of HDAC6 and indirectly decrease HIF-1α expression via HSP90/HDAC6-HIF-1α pathway. Specifically, HDACi indirectly inhibits HSP90 through hyperacetylation of HSP90 and prevents stabilization of corresponding client protein [22]. To date, preclinical studies have demonstrated that combination of HDACi with conventional cytotoxic regimens could be effective for patients with relapsed ovarian cancer, which also resulted in a significant regression of lesion in xenograft model of high grade serous carcinoma (HGSC) [23, 24].

Based on these, there is considerable interest in investigating underlying mechanism of HDACi-induced HIF-1α suppression. In this study, we found that HDACi repressed HIF‐1α expression via inhibiting phosphoinositide 3-kinase (PI3K) and glycogen synthase kinase 3β (GSK3β) pathway. Apart from HSP90 inactivation, HDACi-induced upregulation of HSP70 facilitates HIF‐1α degradation. On the other hand, our study for the first time showed that interaction of HDAC and chaperone system including chaperone HSP70, chaperonin TCP-1α and co-chaperone VHL-binding protein-1 (VBP-1) maintains the stringent quality control of pVHL in ovarian cancer.

## Materials and methods

### Cell culture

Detail of ovarian carcinoma cell lines from previous publications was screened to perform cell lineage tracing, including the primary treatment, histopathology of patients and establishment of cell lines. As a result, the human ovarian cancer cell lines OV-90 and SKOV-3 were finally selected. The OV-90 cell line was derived from ascites of a female with stage IIIc ovarian carcinoma who was sensitive to chemotherapy regimens, and the pathological diagnosis was HGSC [25, 26]. The SKOV-3 cell line was obtained from ascites of a female with ovarian carcinoma who previously received thiotepa only, and the pathological diagnosis was moderately differentiated adenocarcinoma, which can be regarded as non-HGSC [27, 28]. OV-90 cell line (Catalogue number: CC0811) was purchased from CellCook Biotech Corporation (Guangzhou, China). SKOV-3 cell line (Catalogue number: TCHu185) was purchased from the State Key Laboratory of Molecular Biology, Institute of Biochemistry and Cell Biology, Shanghai Institutes for Biological Sciences, Chinese Academy of Sciences (Shanghai, China). SKOV-3 and OV-90 cell lines were grown and maintained in McCoy’s 5A Medium (containing 10% foetal bovine serum, FBS; purchased from Solarbio, China) and in Dulbecco’s Modified Eagle’s Medium (DMEM, containing 15% FBS; purchased from Gibco, Grand Island, NY, USA), respectively. OV-90 and SKOV-3 cells were cultured and generated at 37°C in a humidified atmosphere containing 5% CO_2_ and then subcultured in logarithmic phase in 3×10^6^/mL suspension.

### MTT assay

OV-90 and SKOV-3 cells were seeded at the density of 5×10^4^ cells/well in 96-well microtiter plates and then treated with either 0.1% DMSO as vehicle control or escalating concentrations of panobinostat (10, 25, 50 and 100 nM) for 24 h and 48 h in 37°C, 5% CO_2_ incubator. Panobinostat (LBH589) was purchased from Beyotime Biotechnology, China. Cell viability was then assessed by 3- (4,5-dimethylthiazol-2-yl)-2,5-diphenyltetrazolium bromide (MTT) assay. Then, we put 10 μL MTT in each well and incubated for another 4 h and then assessed the OD levels at 490 nm. The IC_50_ values obtained from *in vitro* experiments were estimated by linear regression analysis of SPSS software, and cell viability was calculated by: $\frac{\mathrm{OD}\;\mathrm{treated}\;\mathrm{well}\;[‐\mathrm{blank}]}{\mathrm{mean}\;\mathrm{OD}\;\mathrm{control}\;\mathrm{well}\;[‐\mathrm{blank}]}\times 100\%$ [29].

### Transfection

OV-90 and SKOV-3 cells were transfected with plasmids and Lipofectamine™ 2000 (Invitrogen, Carlsbad, CA, USA). We acquired plasmids of VHL and VBP-1 from Shanghai GenePharma (Shanghai, China). All gene sequence information was searched from GenBank of National Center for Biotechnology Information (NCBI). OV-90 and SKOV-3 cells (5×10^5^ cells/well) were seeded in 6-well plates and grown to 50% confluence. Cells were divided into 2 groups: the control group and the VHL-VBP-1 co-transfected group. The transfection mixture was replaced after 6 h with DMEM plus 15% FBS for OV-90 cell line and McCoy’s 5A plus 10% FBS for SKOV-3 cell line. Then, the cells were incubated for another 48 h and subjected to western blot analysis.

### Western blot analysis and antibodies

Cell lysates were prepared in 100 μL modified RIPA lysis buffer (containing 1 mM PMSF), and the samples were centrifuged for 10 min at 12,000 rpm, in which supernatant fraction was retained. Total protein was extracted from tumor cells and then the lysates were resolved in SDS/PAGE gels and electrophoretically transferred onto a PVDF membrane (Millipore, Burlington, MA, USA). The membrane was blotted with 5% non-fat milk and washed by PBS containing 0.5% Tween-20. Immunoblotting was performed with the indicated antibodies overnight at 4°C. The probed polyclonal antibodies against HIF-1α (1:500, AF1009), pVHL (1:1000, AF6292), PI3K-p85 (1:1000, AB86714), GSK3β (1:1000, AF5016), TCP-1α (1:1000, DF67174), HSP70 (1:1000, BF0633), and lactate dehydrogenase-A (LDHA) (1:1000, DF6280) were purchased from Affinity (Cincinnati, OH, USA) and Abcam (Cambridge, MA, USA). The anti-β actin antibody (1:1000, TA-09), horseradish peroxidase (HRP)-labeled goat anti-mouse antibody (ZB-2305) and HRP-labeled goat anti-rabbit antibody (ZB-2301) were obtained from ZSGB-BIO Corporation, China. The membranes were then incubated with secondary antibodies after being washed and the above procedures were repeated 3 times. The immunoreactivity was detected and visualized by enhanced chemiluminescence (Thermo Fischer Scientific, Waltham, MA, USA). Then, the membranes were scanned and the images of gray values were calculated with Image J software (National Institute of Mental Health Bethesda, MD), in which quantification analysis was performed by normalizing the gray value of target protein with β-actin for each sample.

### Statistical analysis

All experiments were repeated at least three times independently. Microsoft Office software 2013 (USA) was used to create the artwork and the figures showed representative experimental results. All data were expressed as mean ± standard deviation (SD) and analyzed by SPSS version 20 statistical software (IBM Corporation, Armonk, NY, USA). Data obtained were analyzed using one-way analysis of variance (ANOVA), Bonferroni post-test and independent-sample t test. In particular, three-way ANOVA analysis was conducted to investigate variables of time, concentration and cell type comprehensively. P-values < 0.05 were considered statistically significant.

## Results

### HDACi suppressed cell viability

**Fig 1:**
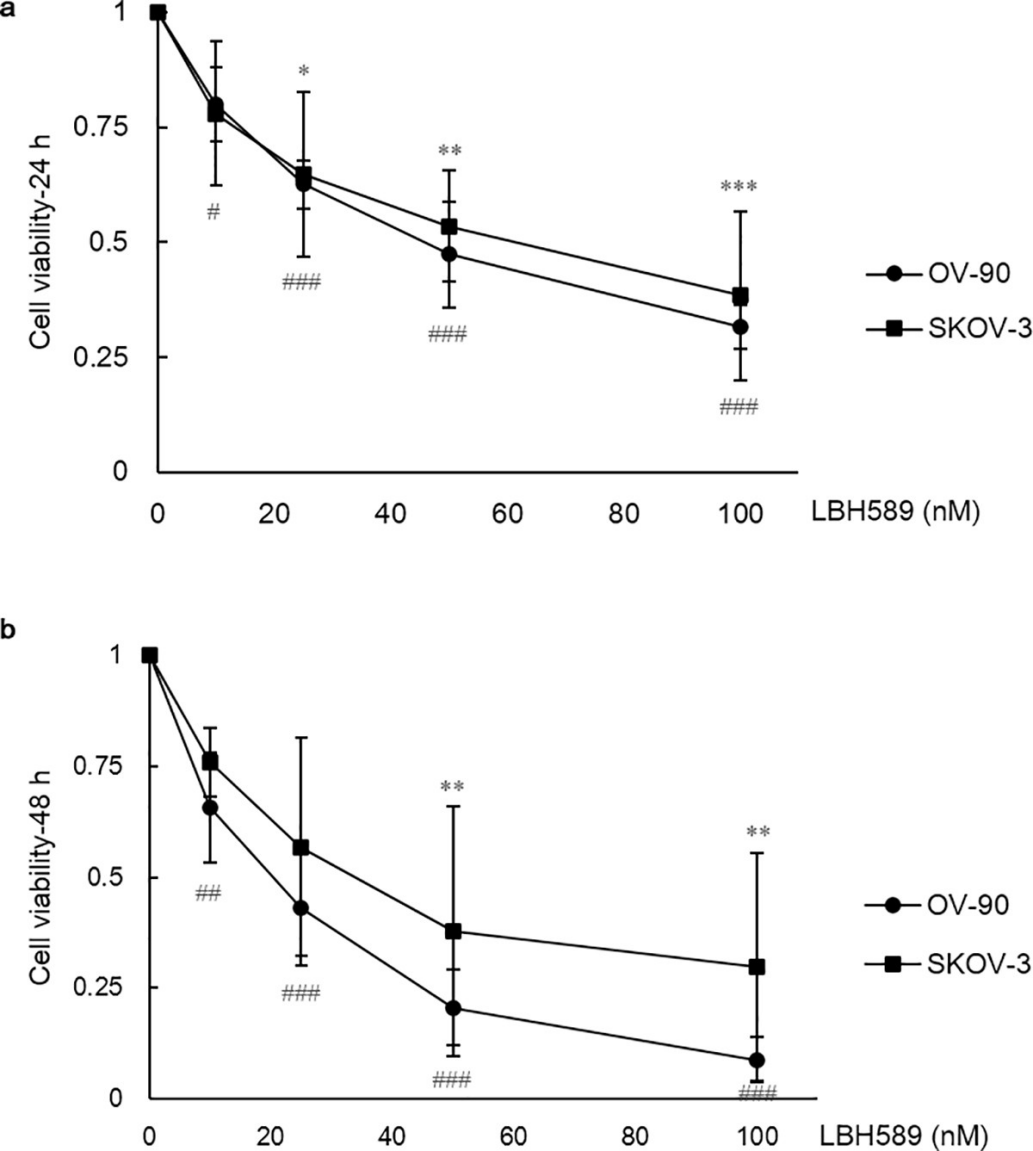
Cellular viability after LBH589 treatment for 24 h and 48 h. LBH589 suppresses cellular viability of OV-90 and SKOV-3 ovarian cancer cell lines. The cell viability was assessed by MTT assay after treatment of LBH589 for 24 h (A) and 48 h (B). Data are shown as mean ± SD of at least three individual experiments (*P < 0.05, **P < 0.01, ***P < 0.001 vs. SKOV-3 control (0.1% DMSO); ^#^P < 0.05, ^##^P < 0.01, ^###^P < 0.001 vs. OV-90 control (0.1% DMSO)).

OV-90 and SKOV-3 ovarian cancer cell lines were treated with increasing concentrations of LBH589 for 24 hours and 48 hours, and cell viability was assessed using MTT assay. The viability of each cell line was drastically reduced in a concentration-dependent fashion when exposed to LBH589 and significant P values were found via one-way ANOVA test and Bonferroni post-test (P = 0.000). Besides, no significant difference of survival was observed in each cell line treated for different length of time (24 h and 48 h). Then we used three-way ANOVA analysis to investigate all three factors including concentration, time and cell type comprehensively. As a result, significant P values were found in groups treated with various concentrations (P = 0.000), groups treated for different length of time (P = 0.000) and groups of different cell types (P < 0.05). Lastly, the IC_50_ of LBH589, defined as a 50% reduction in cell viability caused by the inhibitor, was calculated to be approximately 43.72 and 17.95 nM for OV-90 cells at 24 and 48 h, respectively. In contrast, the SKOV-3 cells were less sensitive to LBH589, with the IC_50_ values being higher (55.98 and 33.56 nM at 24 h and 48 h, respectively). These results are summarized in Fig 1.

### HDACi repressed HIF-1α expression via inhibiting PI3K and GSK3β pathway

**Fig 2:**
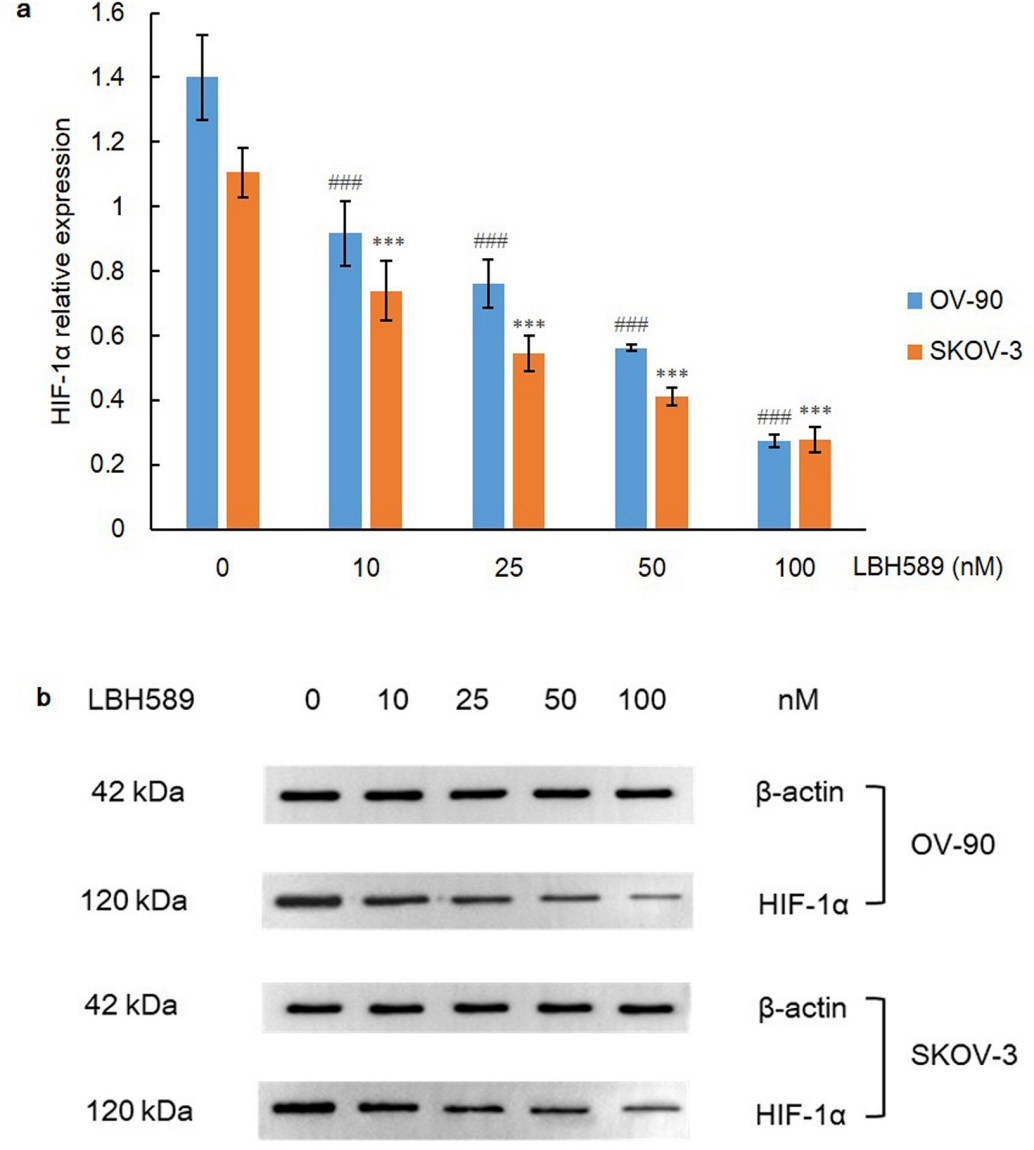
HIF-1α expression after LBH589 treatment for 48 h. A) LBH589 suppresses expression of HIF-1α in OV-90 and SKOV-3 cell lines. Data are shown as mean ± SD of at least three individual experiments. (*P < 0.05, **P < 0.01, ***P < 0.001 vs. SKOV-3 control (0.1% DMSO); ^#^P < 0.05, ^##^P < 0.01, ^###^P < 0.001 vs. OV-90 control (0.1% DMSO)). B) The expression of HIF-1α was assessed by western blot analysis after LBH589 treatment for 48 h. The equal protein loading is shown by detection of β-actin. The figures show representative blots which were cropped from original images.

**Fig 3:**
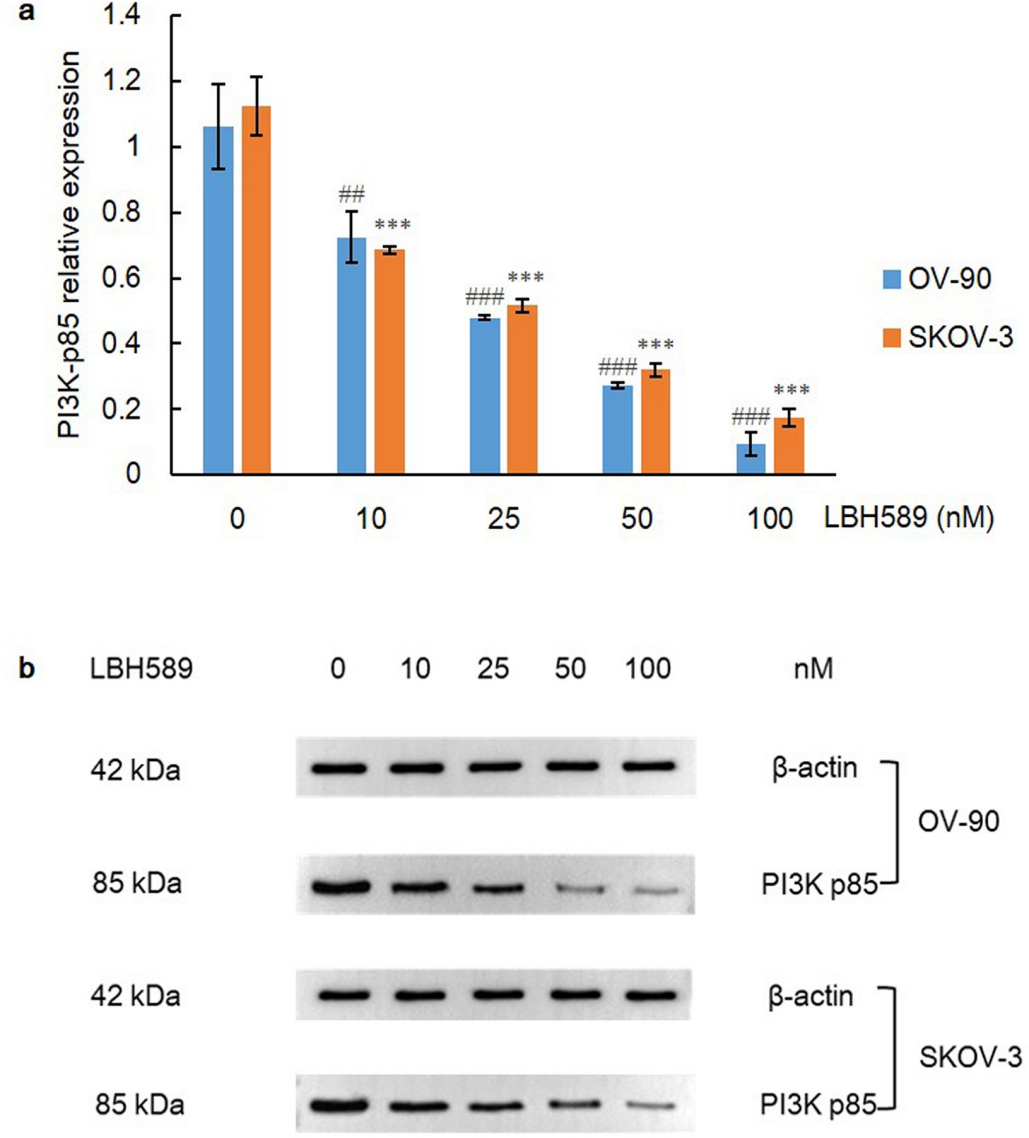
PI3K expression after LBH589 treatment for 48 h. A) LBH589 suppresses expression of PI3K in OV-90 and SKOV-3 cell lines. Data are shown as mean ± SD of at least three individual experiments. (*P < 0.05, **P < 0.01, ***P < 0.001 vs. SKOV-3 control (0.1% DMSO); ^#^P < 0.05, ^##^P < 0.01, ^###^P < 0.001 vs. OV-90 control (0.1% DMSO)). B) The expression of PI3K was assessed by western blot analysis after LBH589 treatment for 48 h. The equal protein loading is shown by detection of β-actin. The figures show representative blots which were cropped from original images.

To determine the outcome of HDACi treatment, the expression of HIF-1α was first determined when OV-90 and SKOV-3 cell lines were treated for 48 h. As a result, HIF-1α level was significantly decreased in each cell line in a dosage-dependent manner (P = 0.000) via one-way ANOVA analysis and Bonferroni post-test (Fig 2). As the upstream molecule of HIF-1α, PI3K plays a pivotal role in up-regulating HIF-1α protein translation via activating Akt and mammalian target of rapamycin (mTOR). mTOR is eventually activated and induces the phosphorylation of ribosomal protein S6 and its complex p70 S6 kinase (S6K) to promote ribosome biosynthesis, thereby promoting hyperactive HIF-1α translation [5]. Concordantly, the expression of PI3K was dramatically declined in both cell lines after exposure to LBH589 for 48 h (P = 0.000) (Fig 3).

**Fig 4:**
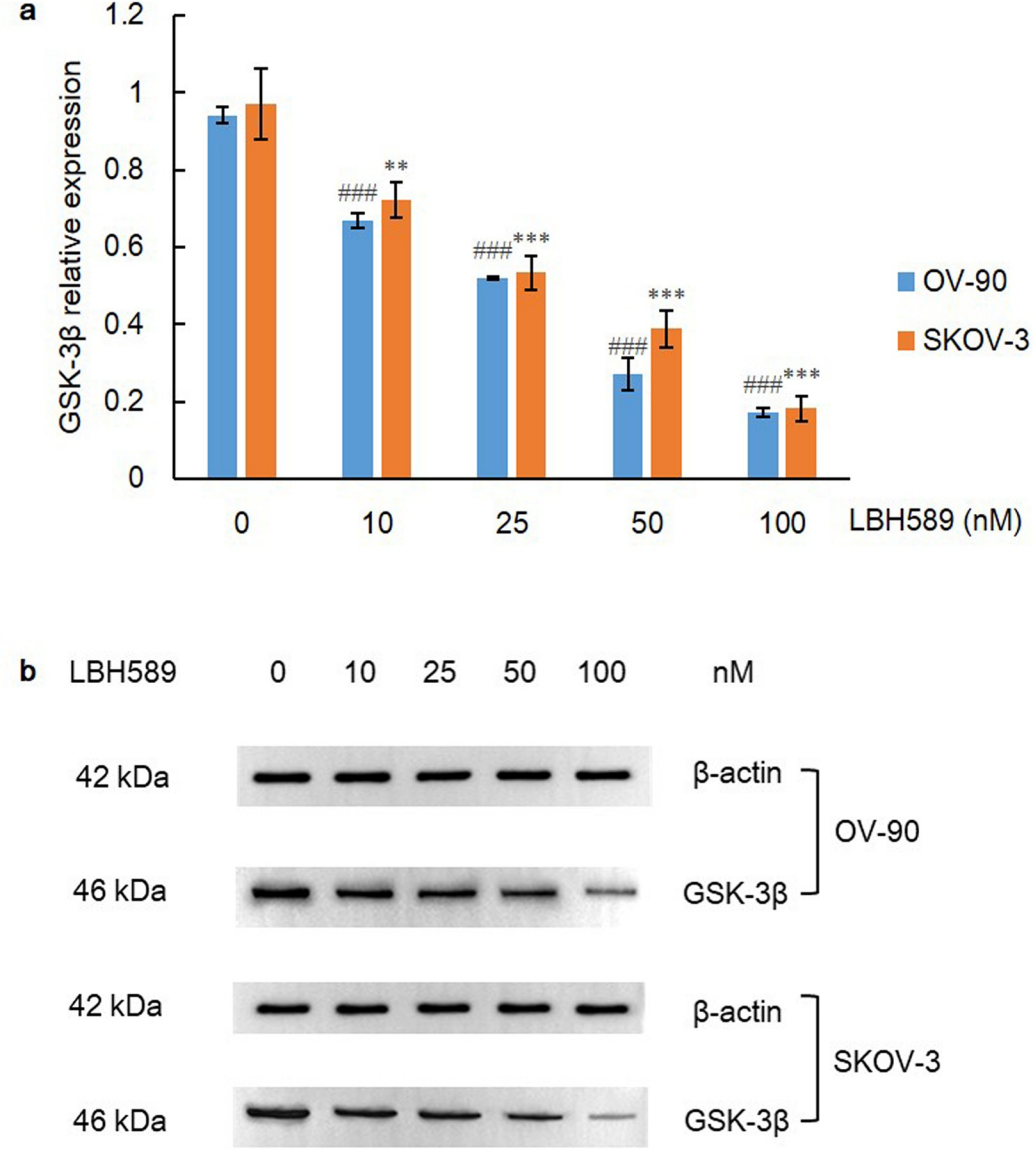
GSK3β expression after LBH589 treatment for 48 h. A) LBH589 suppresses expression of GSK3β in OV-90 and SKOV-3 cell lines. Data are shown as mean ± SD of at least three individual experiments. (*P < 0.05, **P < 0.01, ***P < 0.001 vs. SKOV-3 control (0.1% DMSO); ^#^P < 0.05, ^##^P < 0.01, ^###^P < 0.001 vs. OV-90 control (0.1% DMSO)). B) The expression of GSK3β was assessed by western blot analysis after LBH589 treatment for 48 h. The equal protein loading is shown by detection of β-actin. The figures show representative blots which were cropped from original images.

GSK3β is a serine-threonine kinase and is involved in the Wnt/β-catenin signaling, which is also a downstream effector of PI3K/Akt pathway [30]. GSK3β acts as a molecular regulation switch while the role of its activation in tumor progression still remains a controversy. On the one hand, upon the stimulus by Wnt in hypoxia, the GSK3/casein kinase 1 (CK1)/Axin/adenomatous polyposis coli (APC) destruction complex in the cytoplasm disintegrates, and the unphosphorylated β-catenin translocates to the nucleus and recruits on HREs, which interacts physically with HIF-1 to promote cell survival and adaptation to hypoxia [31]. Additionally, a previous study revealed overexpression of GSK3β inhibited β-catenin and HIF-1α expression [32]. On the other hand, both β-catenin and GSK3β were highly expressed in ovarian cancer [33]. Treatment of PI3K-α inhibitor simultaneously repressed phosphorylation level of Akt, mTOR and GSK3β with a decreased expression of HIF-1α [34]. In this study, we also measured the expression level of GSK3β, and a dramatic downregulation was found in both cell lines (P = 0.000) (Fig 4).

### HDACi-induced pVHL independent degradation of HIF-1α

**Fig 5:**
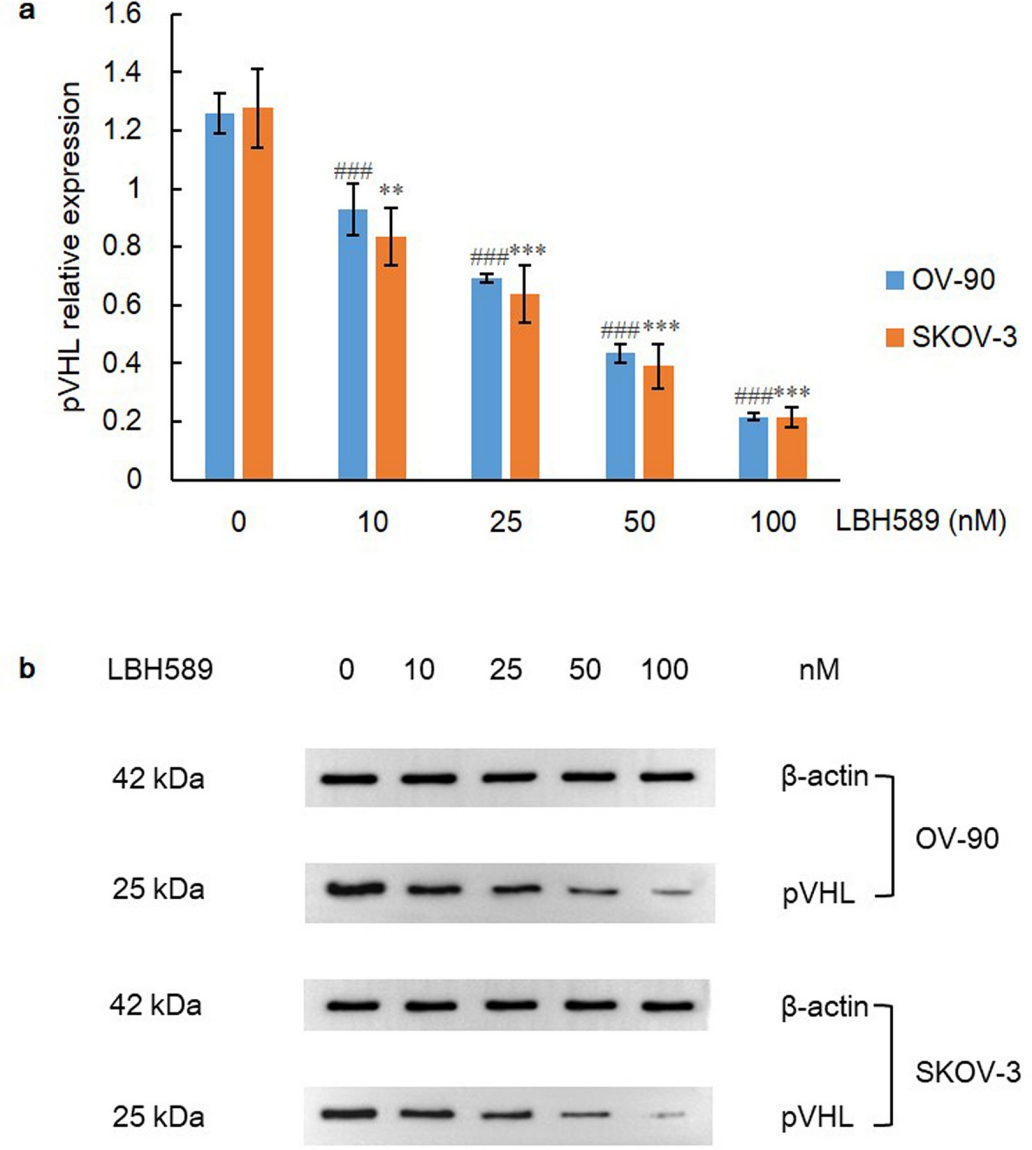
pVHL expression after LBH589 treatment for 48 h. A) LBH589 inhibits expression of pVHL in OV-90 and SKOV-3 cell lines. Data are shown as mean ± SD of at least three individual experiments. (*P < 0.05, **P < 0.01, ***P < 0.001 vs. SKOV-3 control (0.1% DMSO); ^#^P < 0.05, ^##^P < 0.01, ^###^P < 0.001 vs. OV-90 control (0.1% DMSO)). B) The expression of pVHL was assessed by western blot analysis after LBH589 treatment for 48 h. The equal protein loading is shown by detection of β-actin. The figures show representative blots which were cropped from original images.

**Fig 6:**
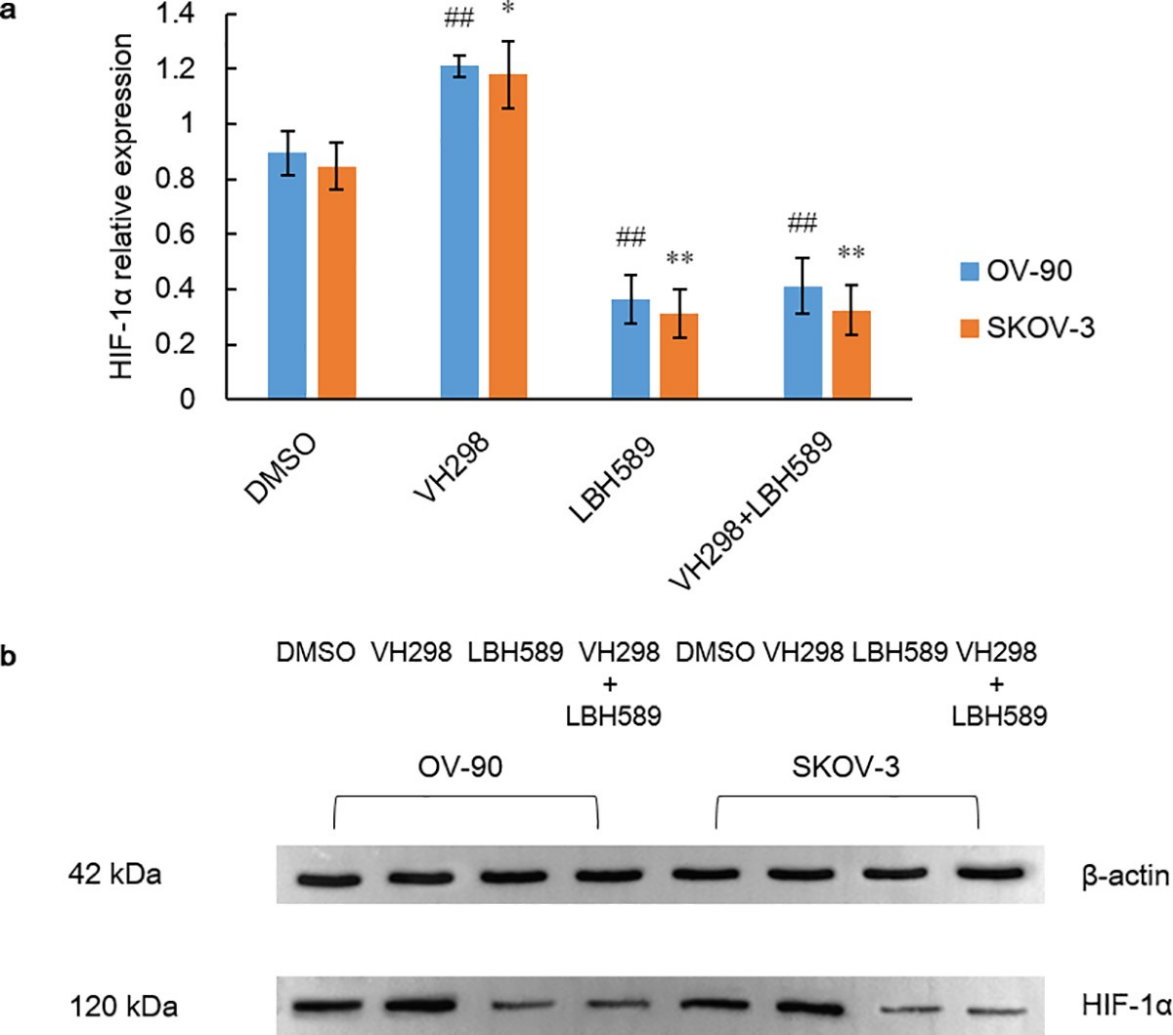
HIF-1α expression after treatment of LBH589 or VH298 for 48 h. A) HIF-1α expressions in OV-90 and SKOV-3 ovarian cancer cell lines were assessed after treatment of DMSO (control), VH298 (10 μM), LBH589 (100 nM), and VH298 (10 μM) + LBH589 (100 nM) for 48 h. Data are shown as mean ± SD of at least three individual experiments. (*P < 0.05, **P < 0.01, ***P < 0.001 vs. SKOV-3 control (0.1% DMSO); ^#^P < 0.05, ^##^P < 0.01, ^###^P < 0.001 vs. OV-90 control (0.1% DMSO)). B) The expression of HIF-1α was assessed by western blot analysis after treatment of DMSO (control), VH298 (10 μM), LBH589 (100 nM), and VH298 (10 μM) + LBH589 (100 nM) for 48 h. The equal protein loading is shown by detection of β-actin. The figures show representative blots which were cropped from original images.

HDACi contributes to hyperacetylation of HSP90 with the repression of chaperone function, leading to the accumulation and vigorous degradation of immature HIF-1α mediated by pVHL [21]. In contrast, the research of Kataria et al. [35] revealed that by targeting the ATP-binding pocket at the N-terminus of HSP90, HSP90 disassociates with HIF-1α, prompting the pVHL-independent degradation of HIF-1α. Therefore, we examine expression level of pVHL to determine whether pVHL directly mediates degradation of HIF-1α upon HDAC inhibition. Intriguingly, a conspicuous declining tendency of pVHL expression was notably shown within the escalating gradient of LBH589 in both cell lines (P = 0.000) (Fig 5). To further support the idea that HDACi-induced degradation of HIF-1α is independent of pVHL, we assessed and compared the expressions of HIF-1α among groups of VHL inhibition (VH289, 10 μM; purchased from Cambridge, USA), HDAC inhibition (LBH589, 100 nM), HDAC plus VHL co-inhibition (VH298, 10 μM plus LBH589, 100 nM) and vehicle control (0.1% DMSO) for 48 h. As a result, HIF-1α expressions were significantly different in group of HDAC inhibition and group of VHL inhibition as compared to control group (P < 0.05) via independent-sample t test. However, no remarkable difference was found between group of HDAC inhibition and group of HDAC plus VHL co-inhibition, suggesting that HDACi-induced HIF-1α degradation may be completely independent of pVHL and that certain chaperone molecules may even participate in the mediation of proteasome degradation of pVHL (Fig 6).

**Fig 7:**
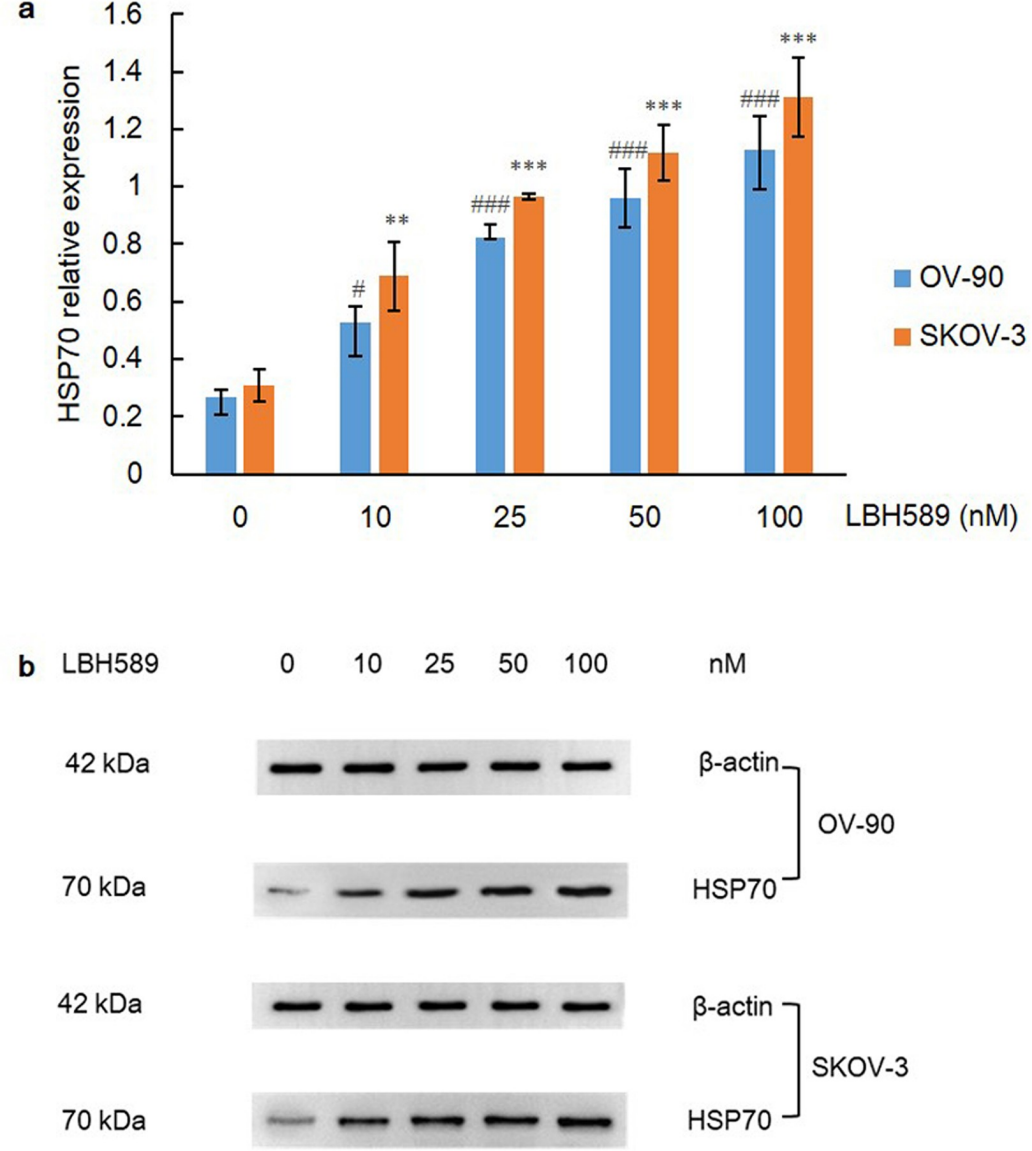
HSP70 expression after LBH589 treatment for 48 h. A) LBH589 promotes expression of HSP70 in OV-90 and SKOV-3 cell lines. Data are shown as mean ± SD of at least three individual experiments. (*P < 0.05, **P < 0.01, ***P < 0.001 vs. SKOV-3 control (0.1% DMSO); ^#^P < 0.05, ^##^P < 0.01, ^###^P < 0.001 vs. OV-90 control (0.1% DMSO)). B) The expression of HSP70 was assessed by western blot analysis after LBH589 treatment for 48 h. The equal protein loading is shown by detection of β-actin. The figures show representative blots which were cropped from original images.

Apart from HSP90, HSP70 not only facilitates nascent protein folding, but also participates in protein degradation, and previous publications have revealed that HIF-1α can be co-immunoprecipitated with HSP90/HSP70 as well as proven the direct binding of HSP70 to the oxygen-dependent degradation domain (ODDD) of HIF-1α [36, 37]. Thus the expression level of chaperone HSP70 was detected and a remarkable increase of HSP70 within escalating concentration of LBH589 was observed in this research (P = 0.000) (Fig 7). Taken together, these data reveal that HSP70 instead of pVHL plays a major role in mediating the degradation of HIF-1α, especially under the condition of HDACi-induced HSP90 inhibition.

### Sophisticated interaction of HDAC and chaperone system in pVHL quality control

**Fig 8:**
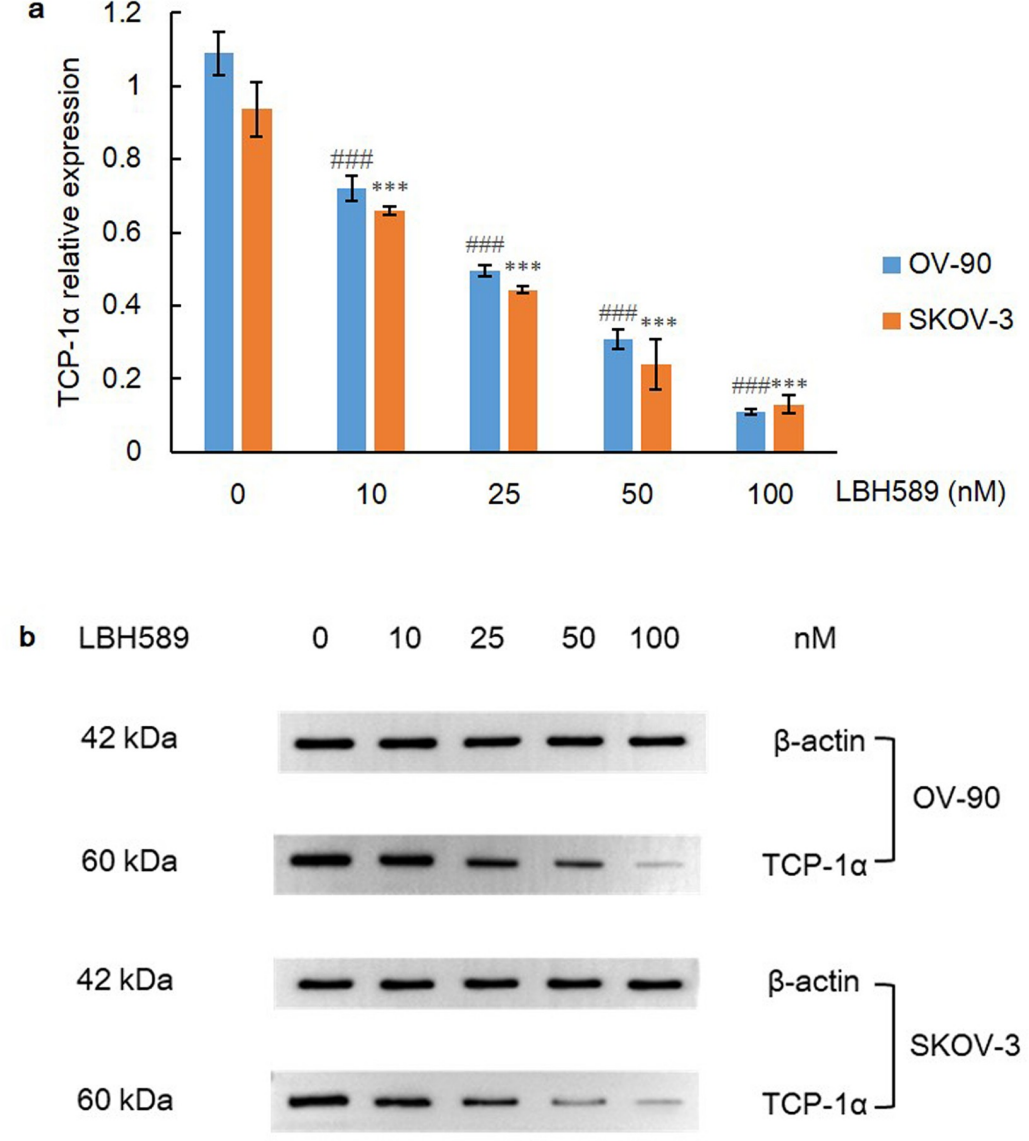
TCP-1α expression after LBH589 treatment for 48 h. A) LBH589 suppresses expression of TCP-1α in OV-90 and SKOV-3 cell lines. Data are shown as mean ± SD of at least three individual experiments. (*P < 0.05, **P < 0.01, ***P < 0.001 vs. SKOV-3 control (0.1% DMSO); ^#^P < 0.05, ^##^P < 0.01, ^###^P < 0.001 vs. OV-90 control (0.1% DMSO)). B) The expression of TCP-1α was assessed by western blot analysis after LBH589 treatment for 48 h. The equal protein loading is shown by detection of β-actin. The figures show representative blots which were cropped from original images.

The stability and function of mature pVHL rely on a sophisticated chaperone machine, and it is now known that chaperonin TCP1 ring complex (TRiC) mediates folding of newly synthesized pVHL [6]. In addition, apart from promoting nascent pVHL folding, HSP70 also plays a major role in degradation of misfolded pVHL, whereas HSP90 is not required for this [38]. Moreover, TCP-1 subunits and prefoldin proteins have been identified as interacting partners that showed high affinity with HDAC1 [39]. Therefore, we hypothesized that HSP70 may be further involved in pVHL degradation when TRiC function is repressed by HDACi. Most importantly, chaperonin TCP-1α, the crucial subunit of TRiC, was conversely reduced in a clear manner in this research (P = 0.000) (Fig 8).

**Fig 9:**
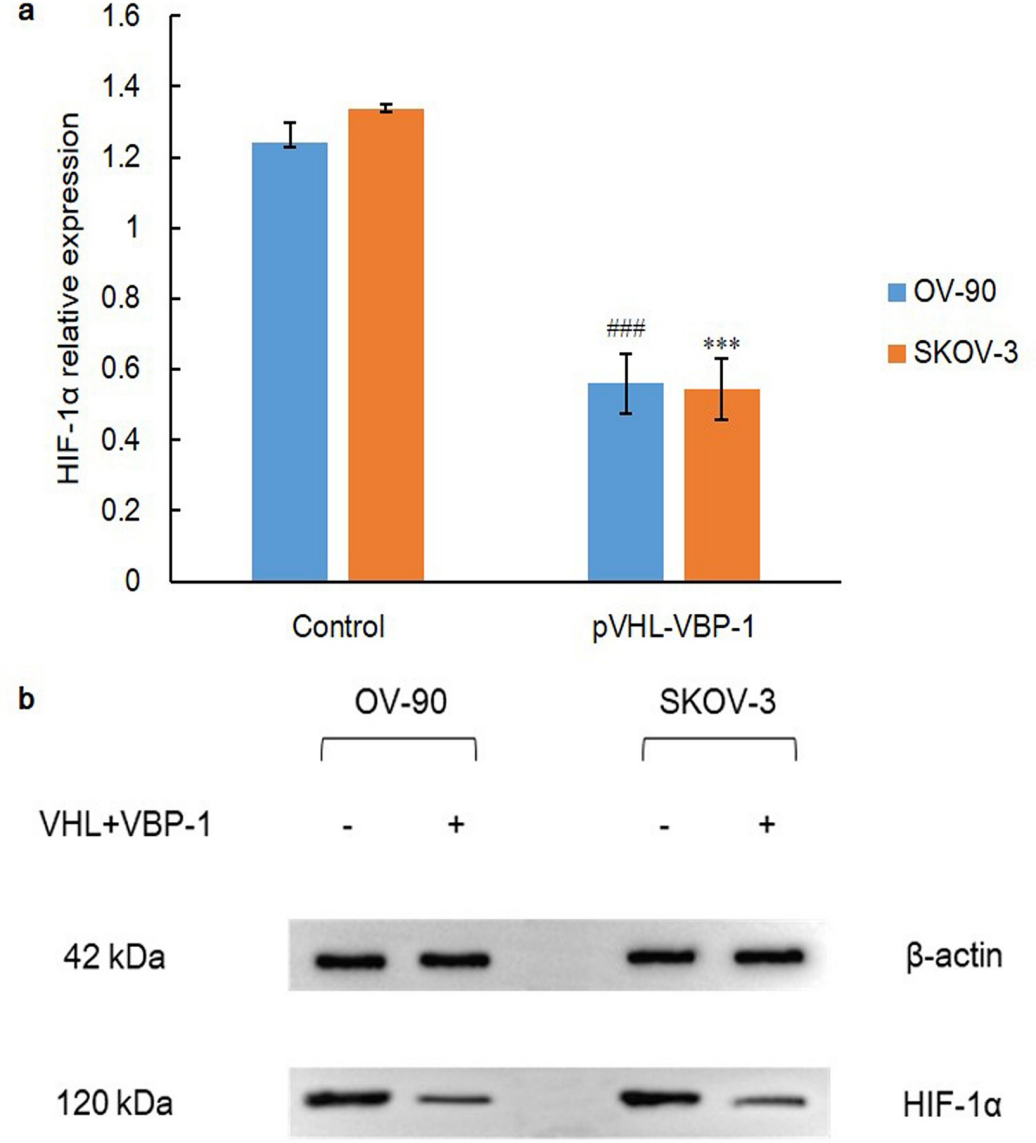
HIF-1α expression after plasmids transfection for 48 h. A) Over-expression of pVHL-VBP-1 inhibits protein expression of HIF-1α in OV-90 and SKOV-3 ovarian cancer cell lines. Data are shown as mean ± SD of at least three individual experiments. (*P < 0.05, **P < 0.01, ***P < 0.001 vs. SKOV-3 control; ^#^P < 0.05, ^##^P < 0.01, ^###^P < 0.001 vs. OV-90 control). B) The expression of HIF-1α was assessed by western blot analysis after transfection of plasmids of VHL and VBP-1 for 48 h. The equal protein loading is shown by detection of β-actin. The figures show representative blots which were cropped from original images.

**Fig 10:**
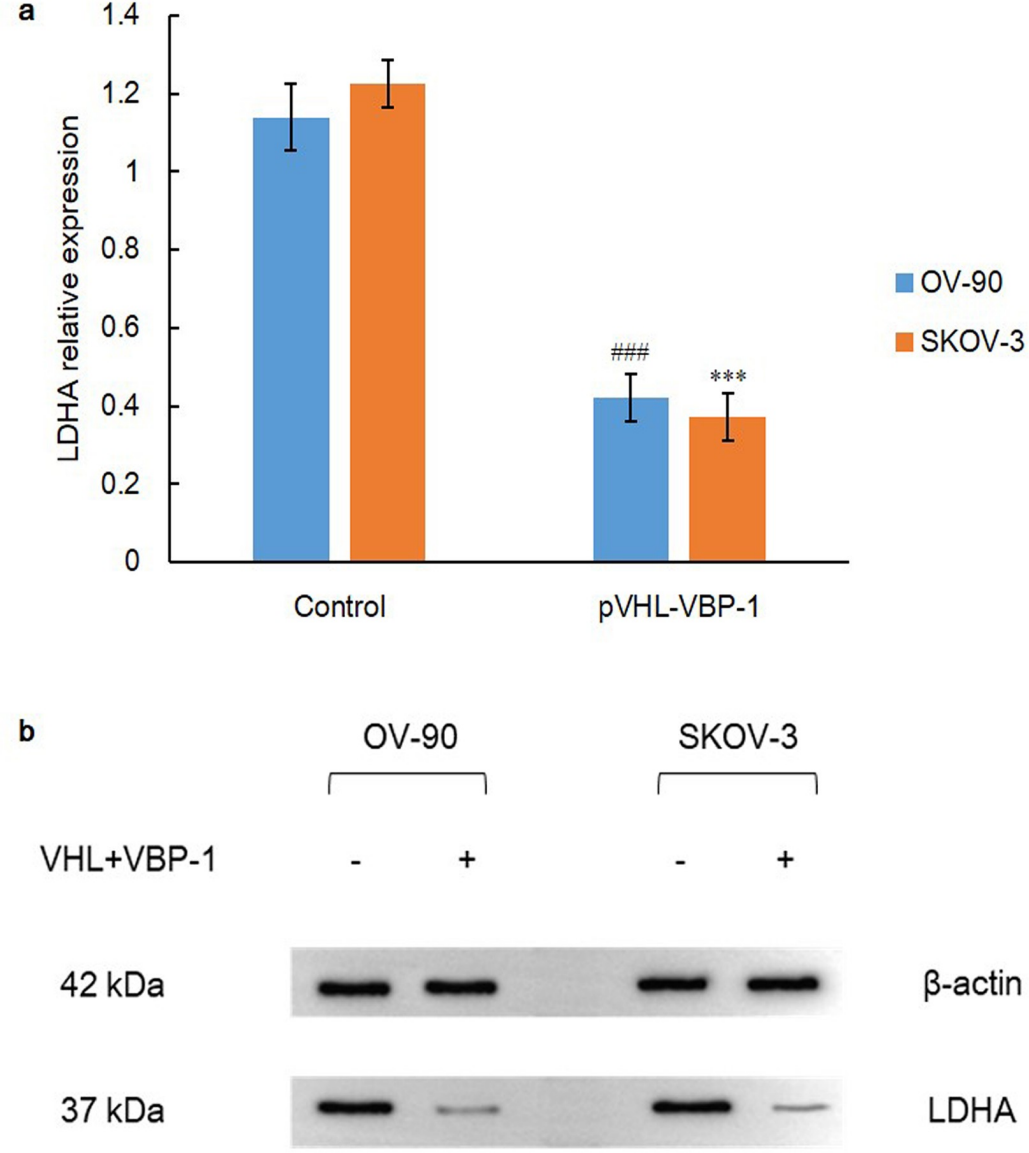
LDHA expression after plasmids transfection for 48 h. A) Over-expression of pVHL-VBP-1 inhibits protein expression of LDHA in OV-90 and SKOV-3 ovarian cancer cell lines. Data are shown as mean ± SD of at least three individual experiments. (*P < 0.05, **P < 0.01, ***P < 0.001 vs. SKOV-3 control; ^#^P < 0.05, ^##^P < 0.01, ^###^P < 0.001 vs. OV-90 control). B) The expression of LDHA was assessed by western blot analysis after transfection of plasmids of VHL and VBP-1 for 48 h. The equal protein loading is shown by detection of β-actin. The figures show representative blots which were cropped from original images.

On the other hand, VBP-1, also known as prefoldin 3, cooperates with TRiC to promote folding of various newly synthesized protein. VBP-1 binds to the C-terminus of pVHL to maintain its stability and avoid being degraded, which further enhances regulatory function of pVHL [40]. Therefore, both cell lines were transfected with VHL plus VBP-1 plasmids for 48 h and we determined the expression status of HIF-1α. As a result, HIF-1α expression was dramatically down-regulated when compared with control group via independent-sample t test (P = 0.000), which again demonstrated that pVHL and prefoldin VBP-1 interaction facilitated HIF-1α degradation remarkably (Fig 9). Besides, as the critical enzyme for lactate degeneration and anerobic glycolysis, LDHA was also found with a drastically decreased expression in co-transfected group through independent-sample t test (P = 0.000) (Fig 10). Collectively, these data demonstrate a stringent network of HDAC and chaperone system in pVHL quality control.

## Discussion

HIF-1 is a heterodimer consisting of α subunit and β subunit, and α subunit is a rudimentary transcriptional factor that responds to the hypoxic stress and senses changes in the oxygen partial pressure. The N-terminus of α subunit contains basic helix-loop-helix (bHLH) family protein and PAS domain, which is involved in dimerization of α/β subunits and DNA binding [36, 41]. The carboxy-terminal transactivation domain (C-TAD) of HIF-1α has a high affinity for binding co-activator CREBB-binding protein (CBP)/p300 and modulates transcriptional activation of HIF-1α [5, 42]. The ODDD on C-terminus of α subunit is responsible for mediating ubiquitin-proteasomal degradation of HIF-1α. In normoxia, the proline residues in the ODDD can be hydroxylated by prolyl-hydroxylase (PHD) whereas the lysine residues in ODDD can be acetylated by the enzyme arrest-defective-1 (ARD-1). This post-translational modification of HIF-1α leads to its recognition by pVHL and tags HIF-1α for degradation [5, 43]. pVHL is coupled with Rbx1, cullin-2, elongin B and elongin C to assemble E3 ubiquitin ligase complex, which mediates ubiquitin-proteasome degradation of HIF-1α [44]. However, the activities of both PHD and ARD-1 are significantly decreased in hypoxia, thereby suppressing the primary oxygen-dependent hydroxylation and acetylation of HIF-1α [5]. Additionally, due to the mutation of p53 and exacerbated binding of HIF-1α and p53 in hypoxia, the stability of HIF-1α increases and the fate of hypoxia-induced apoptosis is prevented, which may contribute to the non-anoikis of the distant metastasis [45, 46].

On the other hand, the hyperactive proliferation of tumors may open the prelude of cell metabolic reprogramming and Warburg in the 1920s postulated that the main source of ATP production switched from oxidative phosphorylation to glycolysis in cellular bioenergetics, even in an aerobic environment [47]. HIF-1α activation is directly and indirectly associated with up-regulation of glucose transporters and glycolytic enzymes seen in glycolysis and lactate production, particularly LDH [42, 48]. Lactate, a final product of anerobic glycolysis, accumulates intracellularly owing to blocking of the tricarboxylic acid (TCA) cycle and increased expression of LDH. HIF binds to HRE region on the promoter of LDH and catalyzes pyruvate into lactate, which augments the effect of pyruvate dehydrogenase kinase 1 (PDK-1) on further impeding the conversion of pyruvate into acetyl-CoA for entry into the TCA cycle [42]. Monocarboxylate transporter (MCT) is closely related to lactate-induced HIF-1 activation, which promotes the rate of lactate shuttling [49]. HIF-1α expression also induces upregulation of transmembrane glycoprotein carbon anhydrase IX (CAIX), which is responsible for regulating surrounding pH via hydrogen-ion exchange and disposing acids synthesized in anerobic metabolism [50]. As a result, the acidic environment and lactate accumulation induced by HIF-1α contribute to T cell functional disorder and immune escape [51, 52].

PI3K is a well-known lipid kinase family member and activated PI3K generates phosphatidylinositol (3,4,5)-trisphosphate (PIP3), which acts as a second messenger in binding to phosphoinositide-dependent protein kinase-1 (PDPK-1) and resulting in the phosphorylation of the serine residues of Akt [5, 53]. mTOR is then activated and phosphorylates the eukaryotic translation initiation factor 4E (eIF4E)-binding protein, contributing to the dissociation of eIF4E, which is essentially the mRNA 5ʹ cap-binding protein. The freeing up of eIF4E allows assembly of the translation initiation complex, resulting in the up-regulation of HIF-1α translation and hence its level in the cell [5, 54]. Clinically, the research of Taylor et al. [55] reported that fifty patients with recurrent ovarian, peritoneal, and fallopian tube cancer received everolimus plus bevacizumab chemotherapy, as a result, one patient had complete response, six had partial responses and 35 had stable diseases. Besides, as the downstream molecule of PI3K/Akt, GSK3β participates in multiple signal pathways and its inactivation in hypoxia may contribute to angiogenesis and tumor growth by facilitating HIF-1α [56]. The phosphorylation of HIF-1α by GSK3β, as mediated through PI3K/Akt, results in the decreased stability of HIF-1α. Activated GSK3β also promotes the F-box and WD repeat domain-containing 7 (FBW7)-mediated degradation of HIF-1α through the phosphorylation of its ODDD sites, independently of pVHL [57]. And the research of Jiang et al. [58] showed that activation of the Wnt/β-catenin signaling pathway may be related to HIF-1α-induced epithelial-mesencymal transition. In this study, after LBH589 treatment for 48 h, PI3K and GSK3β expressions were significantly reduced (P = 0.000), which might be attributed to HDACi-induced hyperacetylation. HDAC promotes deacetylation of lysine residues of GSK3β and overexpression of HDAC has been confirmed to upregulate GSK3β expression and activity, while its deficiency impairs the activity of GSK3β to phosphorylate its substrate [59, 60]. In addition, HDAC6 inhibition results in acetylation of Akt and decreases its ability binding PIP3 and phosphorylating downstream molecules [61].

HSP90 upregulation has been proved as a key element for stabilization of HIF-1α in acidic and hypoxic niche, independent of PHD and pVHL [62]. HSP90 promotes rapid accumulation of HIF-1α through the direct interaction with PAS domain of HIF-1α, thereby inducing structural change of HIF-1 heterodimer and stabilizing HIF-1α against pVHL-independent degradation [20, 63]. Kataria et al. [35] revealed that via inhibiting C-terminus of HSP90, proteasome degradation of HIF-1α would be initiated via PHD, even in hypoxia. Another subset of chaperone family member HSP70, is the cytosolic substrate of HDAC5 and HDACi-induced hyperacetylation renders HSP70 a higher affinity to HIF-1α, leading to the accelerated degradation and attenuated nuclear accumulation of HIF-1α [37]. HSP70 recruits and associates with carboxyl terminus of Hsc70-interacting protein (CHIP), which functions as an E3 ubiquitin ligase, mediating proteasome degradation of HIF-1α [64]. Therefore, in this study, both ovarian cell lines were exposed to HDACi with the HSP90 targeting effect and a dramatic decrease of HIF-1α within the increasing gradient of LBH589 was observed (P = 0.000). In addition, no significant difference of HIF-1α expressions was found between groups of single HDAC inhibition and HDAC plus VHL co-inhibition, indicating HDACi-induced HIF-1α degradation is totally independent of pVHL.

It is clear that chaperone system including prefoldin VBP-1, HSP70/90 and TRiC is critical in protein homeostasis, and our result interestingly showed a down-regulation of pVHL expression (P = 0.000). The present literature has revealed that HSP70 cooperates with chaperonin TRiC in correct folding of newly translated pVHL in a sequential manner, which is latter coupled to assembly of trimeric VBC complex (pVHL-Elongin BC) [38, 65]. Specifically, the Box 1 and Box 2 regions of pVHL contain the chaperonin-binding motifs and strengthen TRiC-pVHL binding both directly and stably [65]. In addition, VBP-1 binds to pVHL as a co-chaperone and the function of VBP-1 relies on the presence of pVHL, in which VBP-1 plus pVHL co-expression induces a more dramatic degradation of HIF-1α than pVHL alone [40]. Moreover, HDAC protein series were found to have interactomes with prefoldin and TRiC in human, and imaging revealed that the prefoldin subunit VBP-1 co-localizes with nuclear HDAC1 and constitutive expression of VBP-1 presents with nuclear HDAC1 [66]. Besides, the association of HDAC3 with TRiC has been confirmed, in which the interaction of HDAC3 and TRiC is essential for nuclear hormone receptor corepressor SMRT-mediated activation of HDAC3 [67].

**Fig 11:**
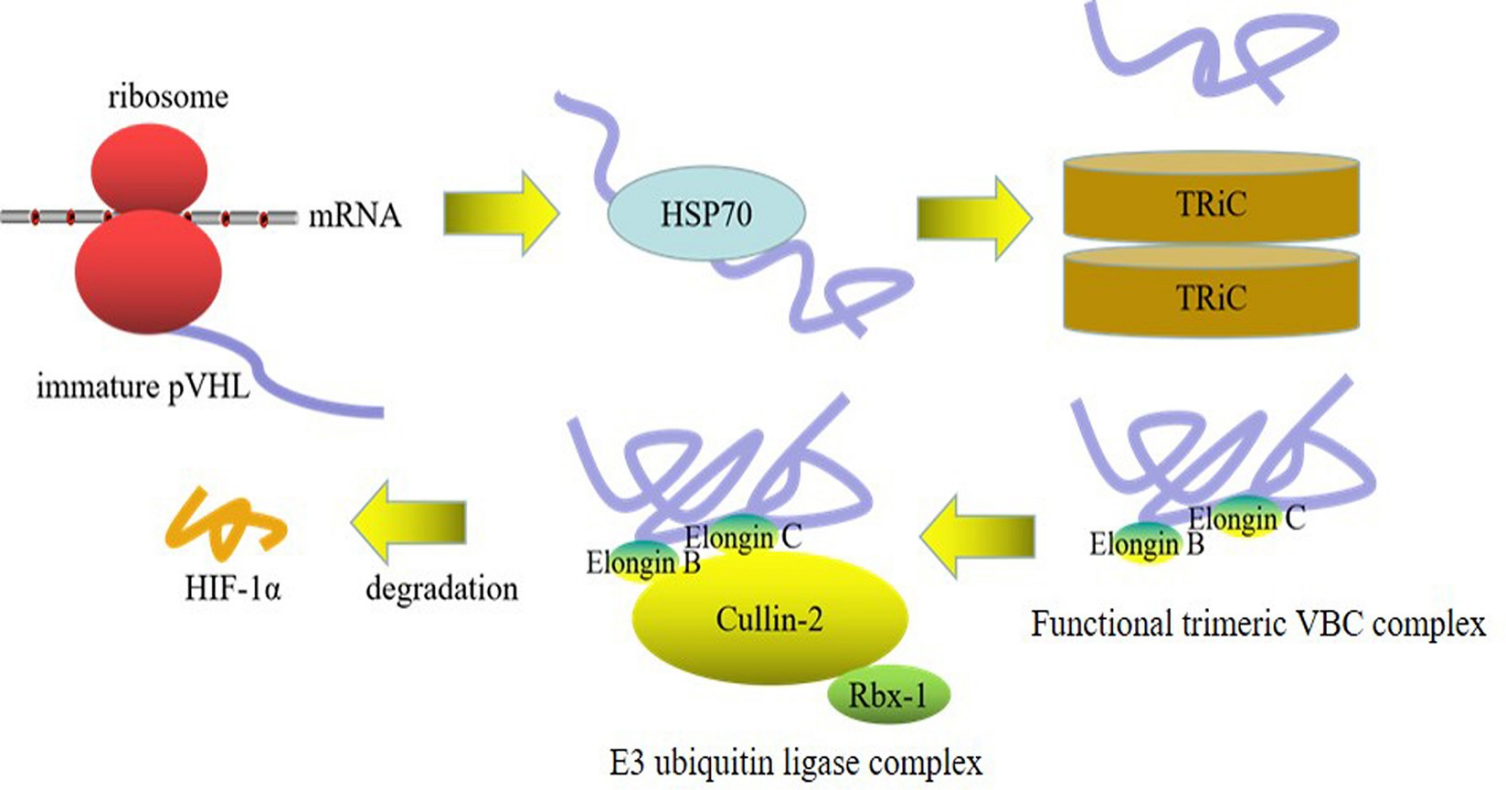
Sequential assembly of VBC complex in HIF-1α degradation. Chaperone HSP70 cooperates with TRiC in correct folding of newly translated pVHL in a sequential manner, which is latter coupled to the assembly of trimeric VBC complex (pVHL-Elongin BC) and mediates degradation of HIF-1α.

**Fig 12:**
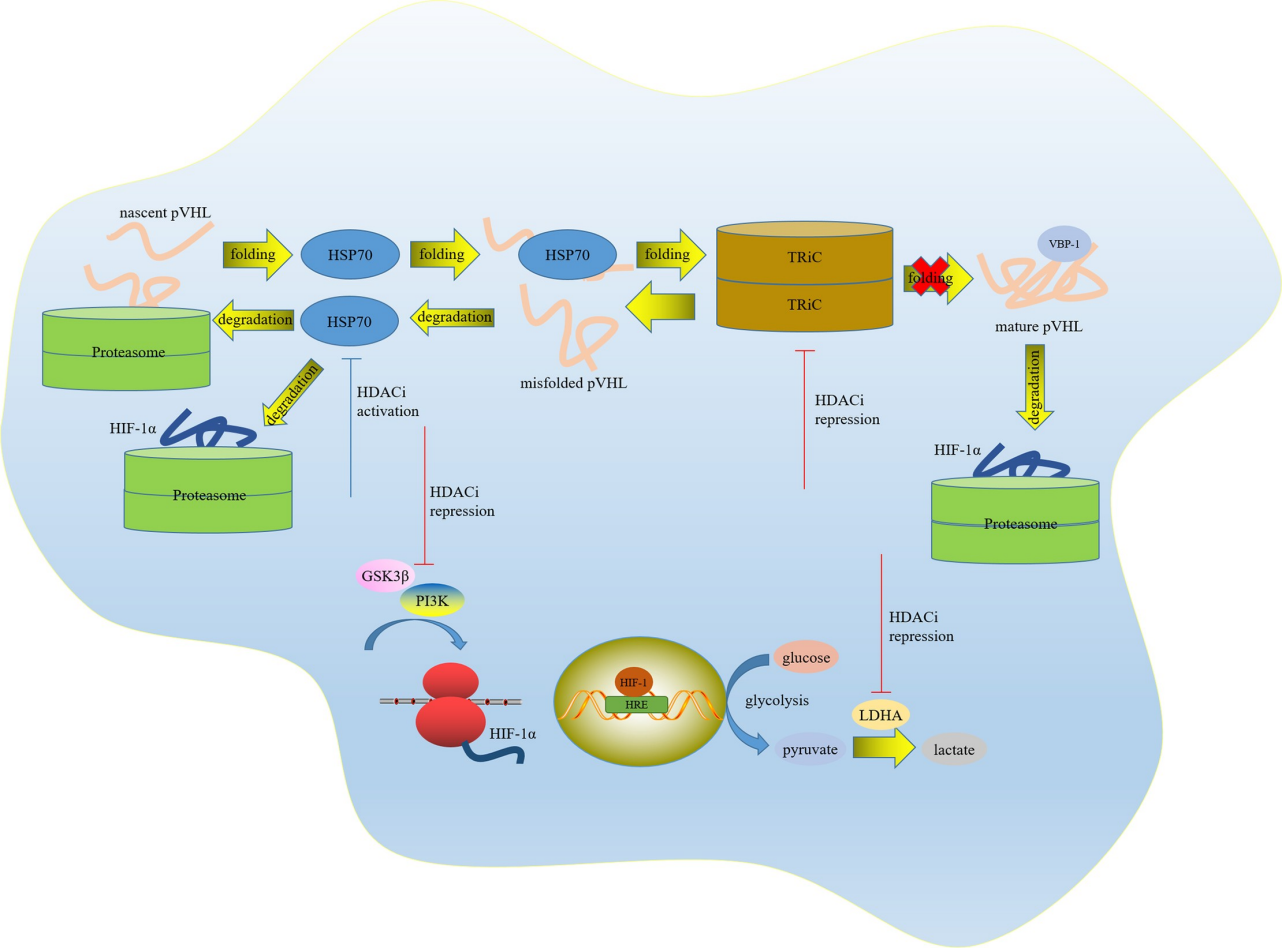
Sophisticated network in pVHL and HIF-1α regulation. HDACi repressed HIF‐1α expression via PI3K and GSK3β and facilitated HIF‐1α degradation via HSP70. The interaction of HDAC and chaperone system maintains the stringent quality control of pVHL.

Therefore, we speculated that the activity of TRiC and the assembly of a functional VBC complex may potentially rely on HDAC status and suppression of HDAC may contribute to dysfunction of chaperonin TRiC and misfolding of pVHL. The conformation instability of pVHL gives rise to its loss of function and degradation, which may be finally resolved by HSP70. Importantly, the western blot results confirmed that the expression level of HSP70 was greatly increased (P = 0.000), and the expression level of TCP-1α was also remarkably declined (P = 0.000) upon HDAC inhibition. Collectively, these data reflect a sophisticated harnessing of the chaperone system, including chaperone HSP70, co-chaperone VBP-1 and chaperonin TRiC, for quality control of pVHL and fine regulation of HIF-1α (Figs 11 and 12).

However, the study has several limitations, on the one hand, *in vitro* cell models may not fully demonstrate heterogeneity of human ovarian cancer. Therefore, future preclinical studies are warranted to testify the anti-tumor potential of HIF-1α axis. On the other hand, this research could develop into more elaborate and sensitive sub-group studies, for example to introduce chemo-resistant cell lines, in this manner, the results could be more convincing and clear.

## Conclusion

On the one hand, treatment with HDACi may decrease HIF-1α gene expression via inhibiting PI3K and GSK3β pathway, and the HDACi-induced pVHL-independent degradation of HIF-1α was actually conducted by HSP70. On the other hand, HDACi-induced dysfunction of TRiC contributes to instability and misfolding of pVHL, and chaperone HSP70 may be responsible for degradation of mutant pVHL, which confirms interaction of HDAC and chaperone system. Prefoldin VBP-1 facilitates function of pVHL as a co-chaperone and VBP-1-pVHL co-expression mitigates glycolysis and lactate metabolism as a consequence. Therefore, all these may indicate that pVHL is finely controlled by a meticulous network of chaperone HSP70, prefoldin VBP-1 and chaperonin TRiC.

## Supporting information

S1 Raw images (PDF)
