# Supplementary material for: Histone deacetylase inhibitor induced pVHL-independent degradation of HIF-1α and hierarchical quality control of pVHL via chaperone system
Source: PLoS One. 2021 Jul 30;16(7):e0248019. doi: 10.1371/journal.pone.0248019 (PMC8323912; doi:10.1371/journal.pone.0248019)
Supplement: S1 Raw images — (PDF) [file pone.0248019.s001.pdf]

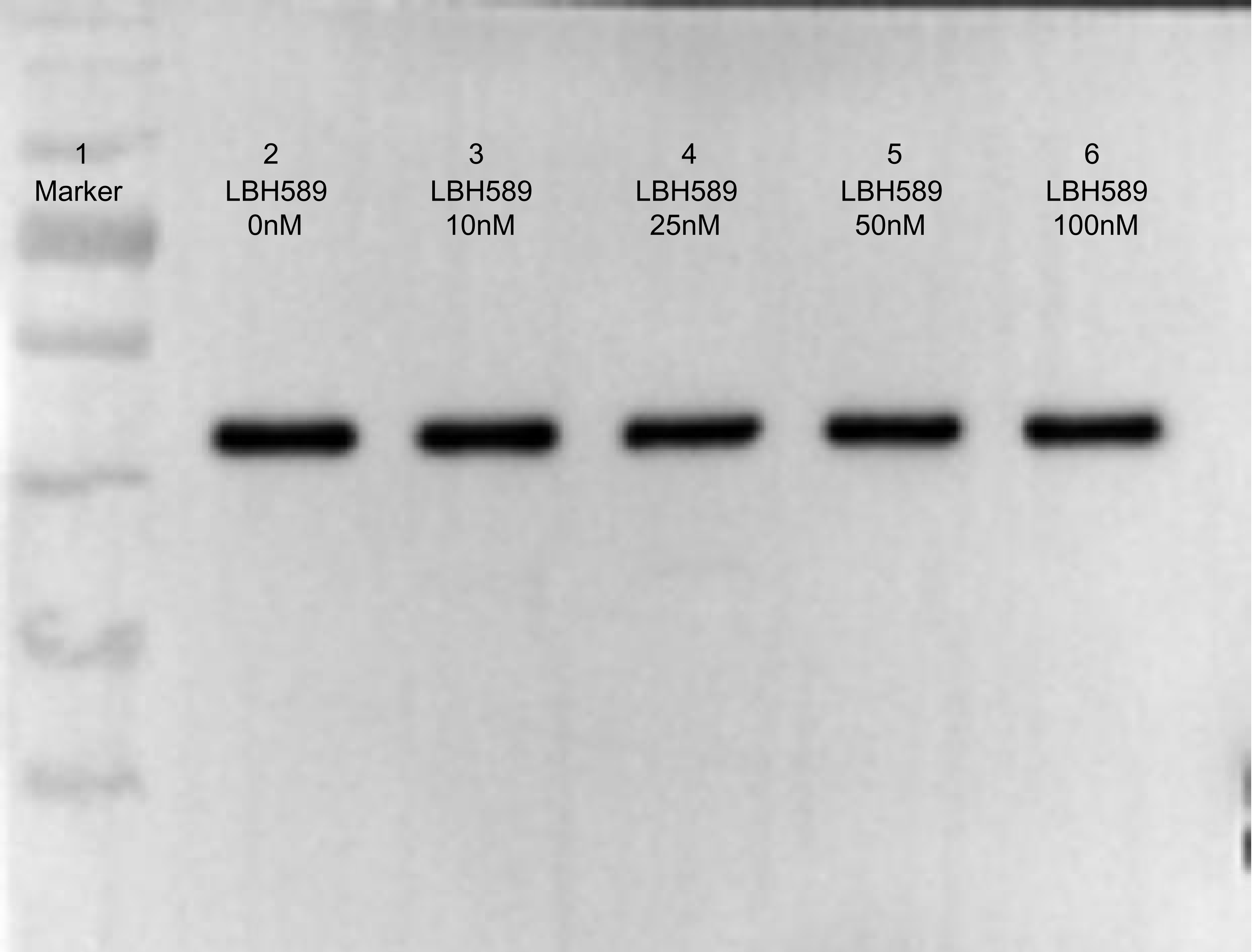

The expression of β-actin in OV-90 was assessed after LBH589 treatment for 48 h. The image was captured by chemiluminescence imaging machine (JS-M6P, Shanghai Peiqing Science & Technology Corporation, China). Fig 2-5 and 7-8 were generated from this original image.

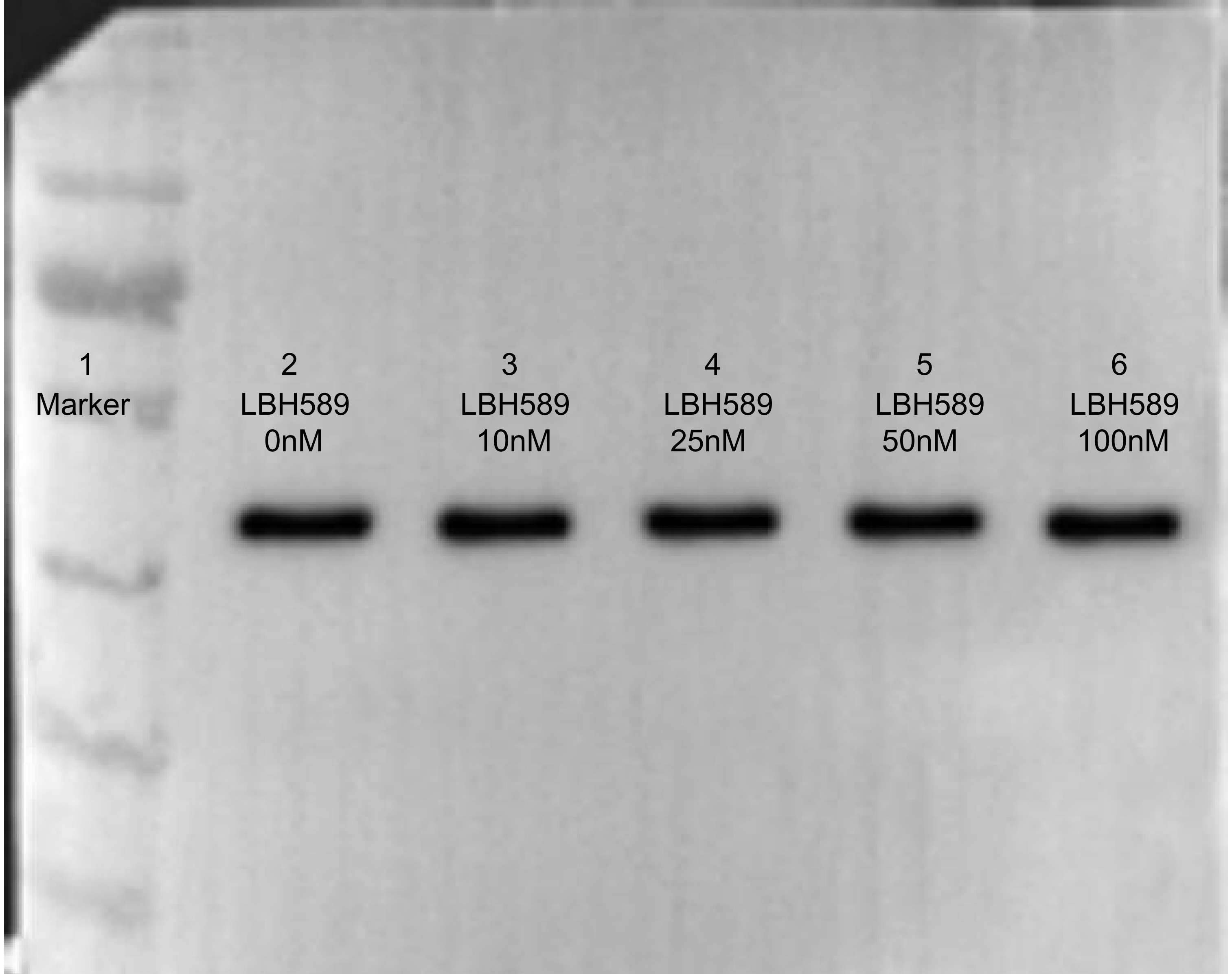

The expression of β-actin in SKOV-3 was assessed after LBH589 treatment for 48 h. The image was captured by chemiluminescence imaging machine (JS-M6P, Shanghai Peiqing Science & Technology Corporation, China). Fig 2-5 and 7-8 were generated from this original image.

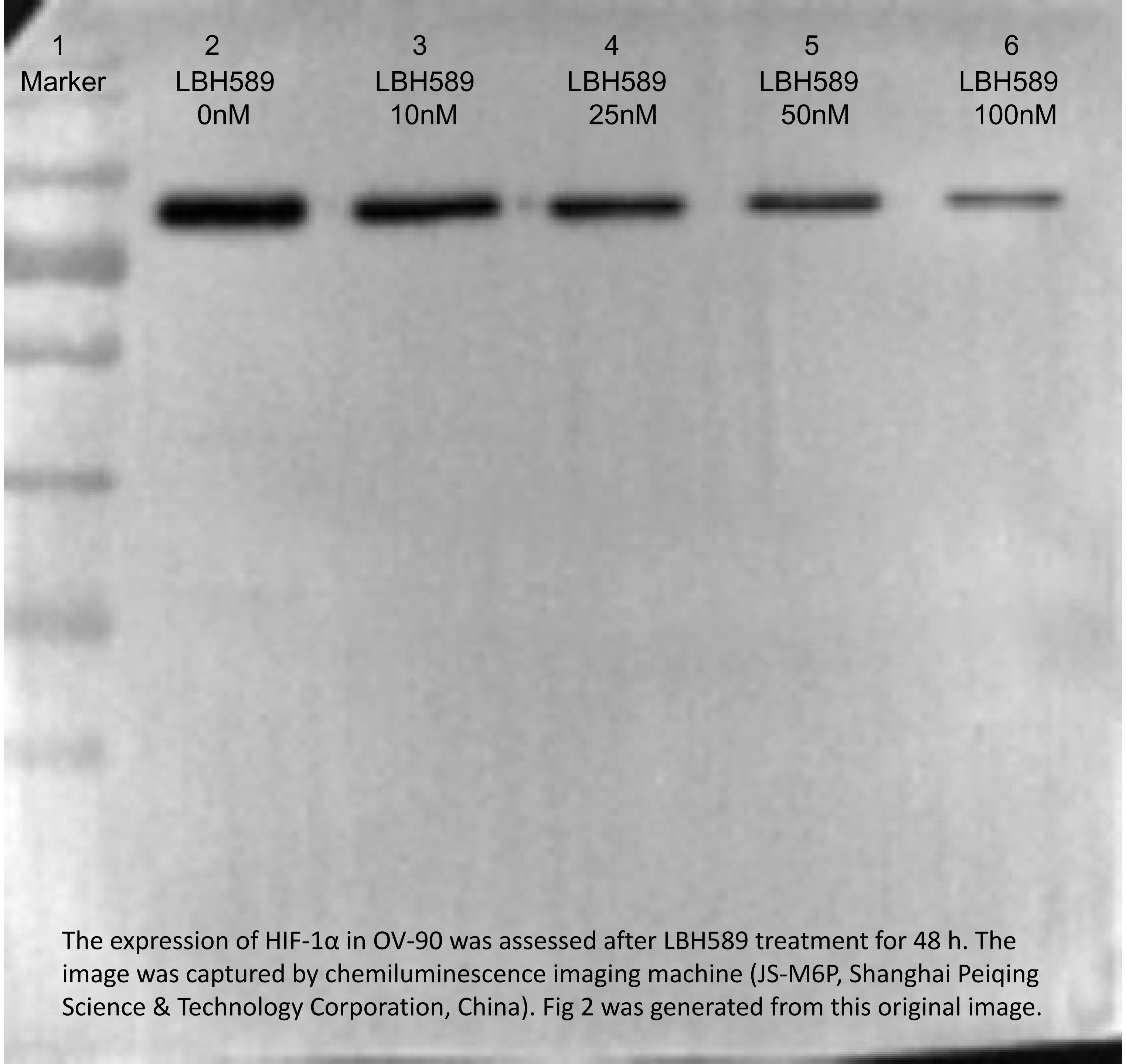

The expression of HIF-1α in OV-90 was assessed after LBH589 treatment for 48 h. The image was captured by chemiluminescence imaging machine (JS-M6P, Shanghai Peiqing Science & Technology Corporation, China). Fig 2 was generated from this original image.

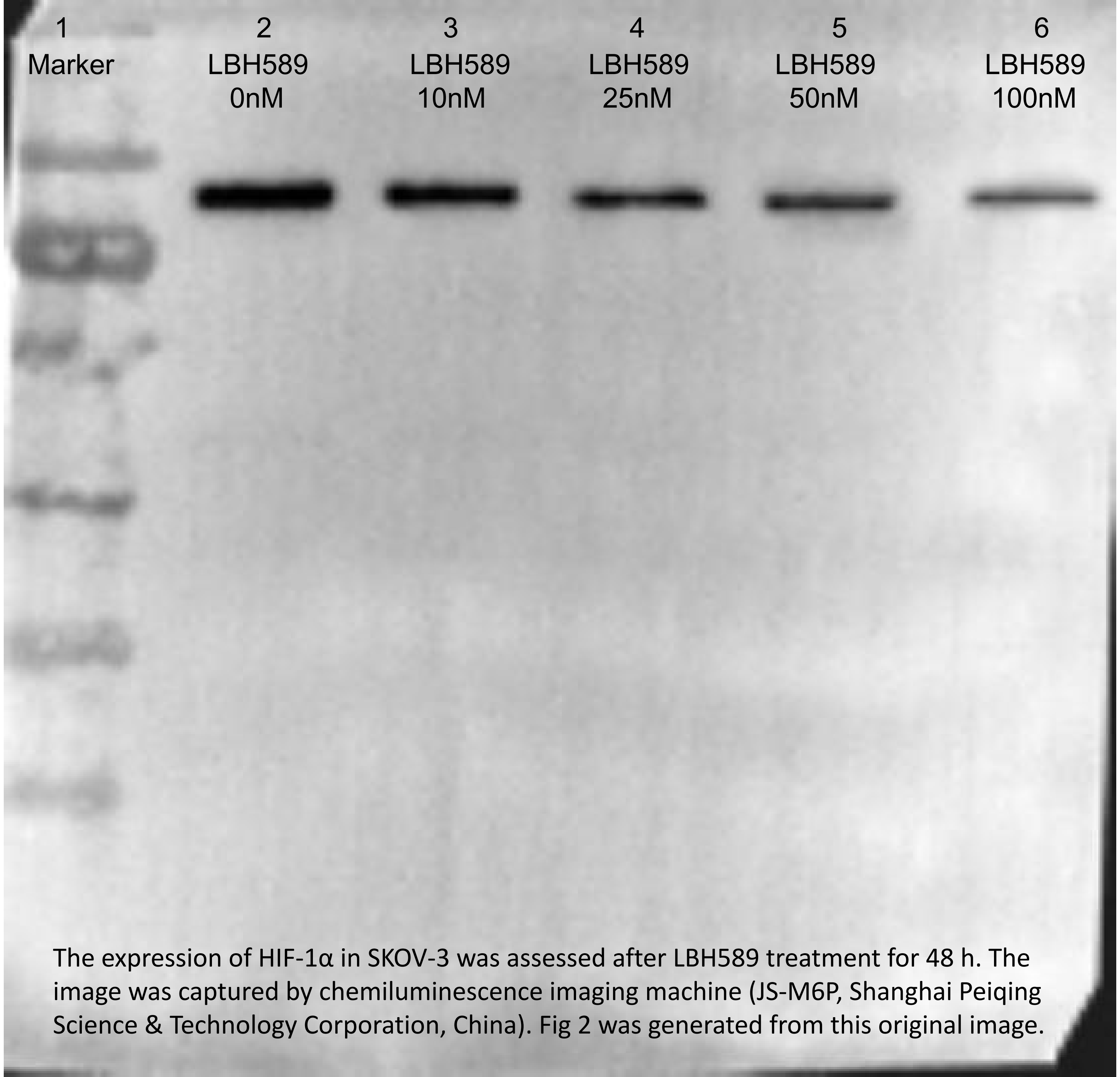

The expression of HIF-1α in SKOV-3 was assessed after LBH589 treatment for 48 h. The image was captured by chemiluminescence imaging machine (JS-M6P, Shanghai Peiqing Science & Technology Corporation, China). Fig 2 was generated from this original image.

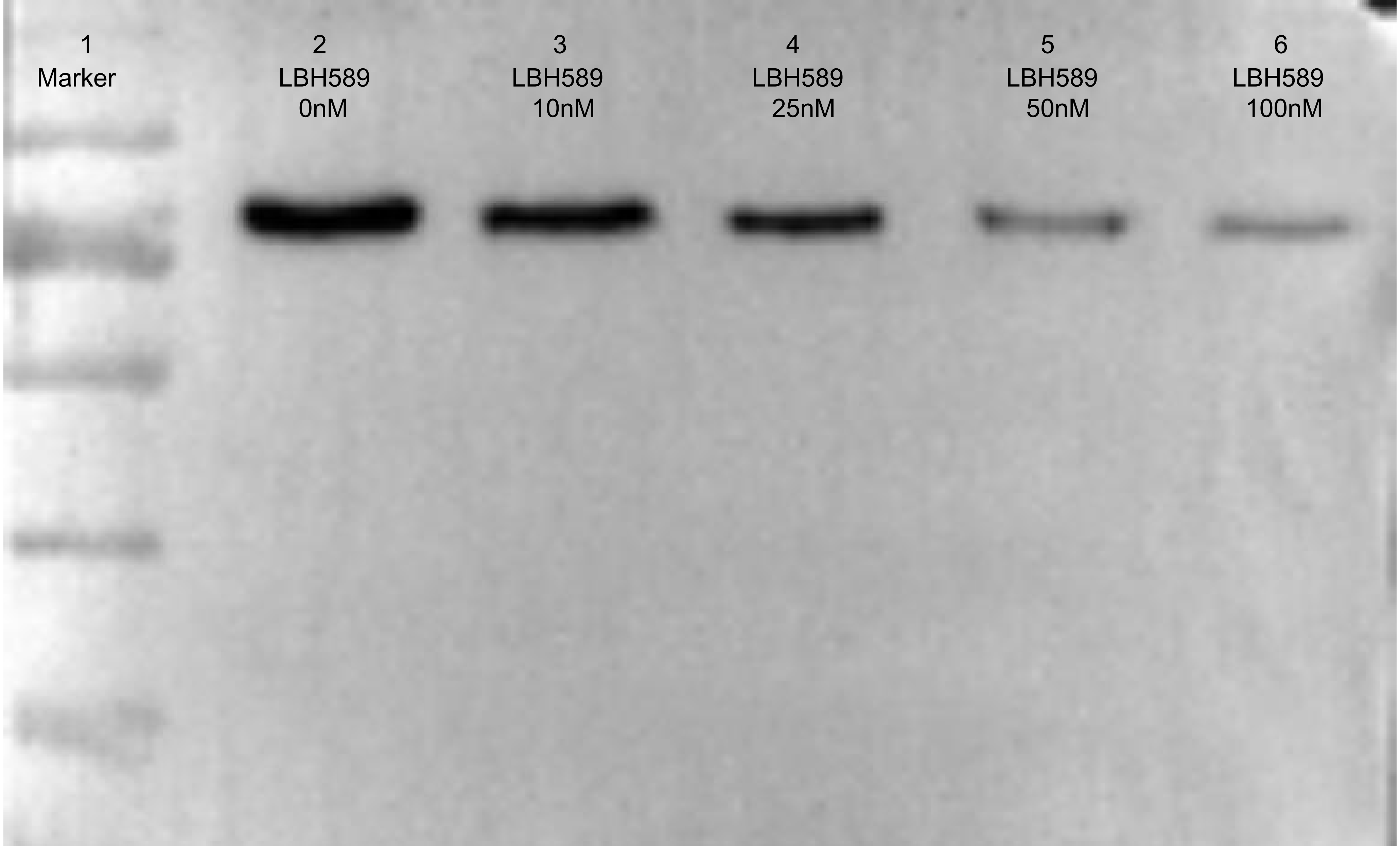

The expression of PI3K in OV-90 was assessed after LBH589 treatment for 48 h. The image was captured by chemiluminescence imaging machine (JS-M6P, Shanghai Peiqing Science & Technology Corporation, China). Fig 3 was generated from this original image.

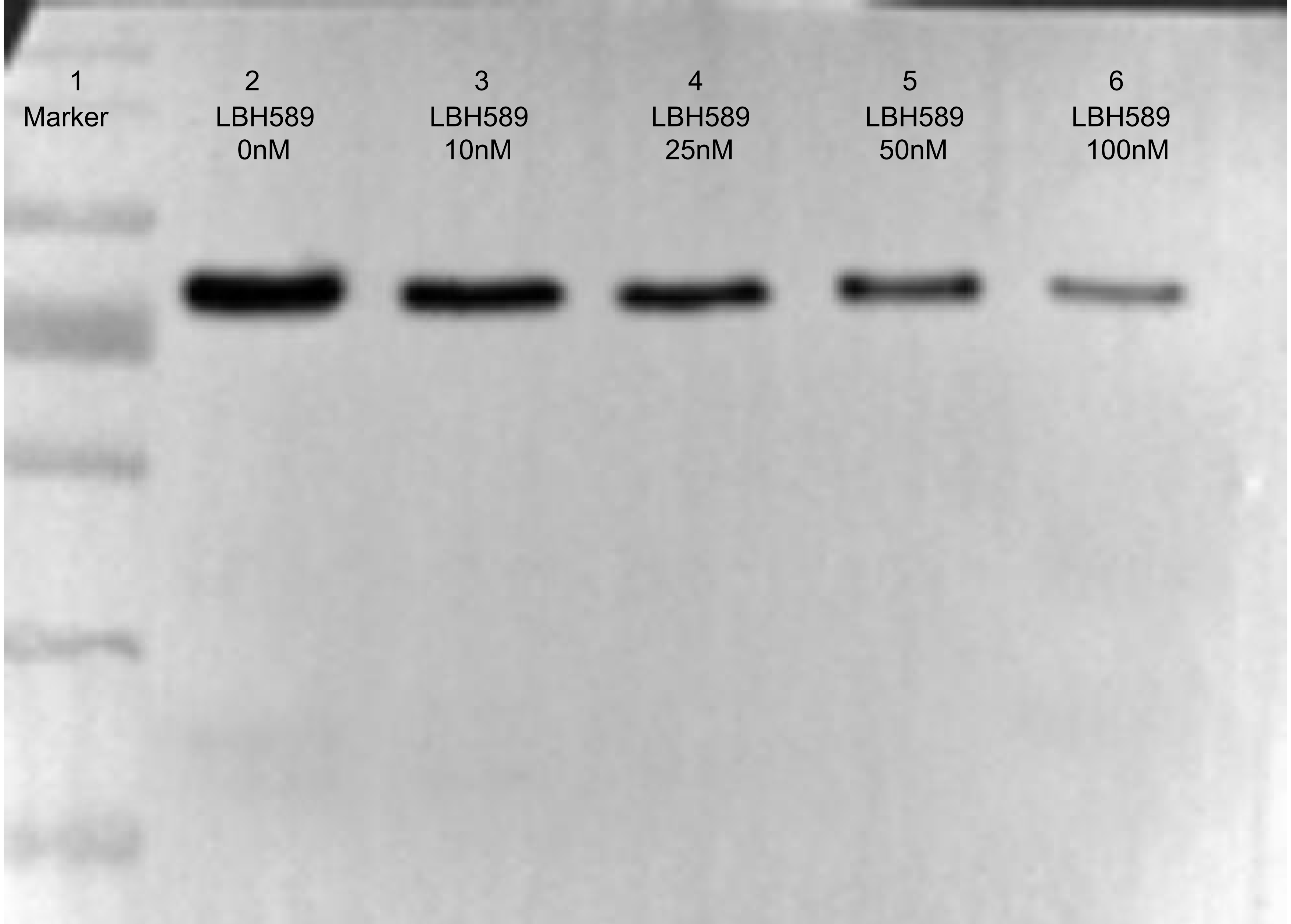

The expression of PI3K in SKOV-3 was assessed after LBH589 treatment for 48 h. The image was captured by chemiluminescence imaging machine (JS-M6P, Shanghai Peiqing Science & Technology Corporation, China). Fig 3 was generated from this original image.

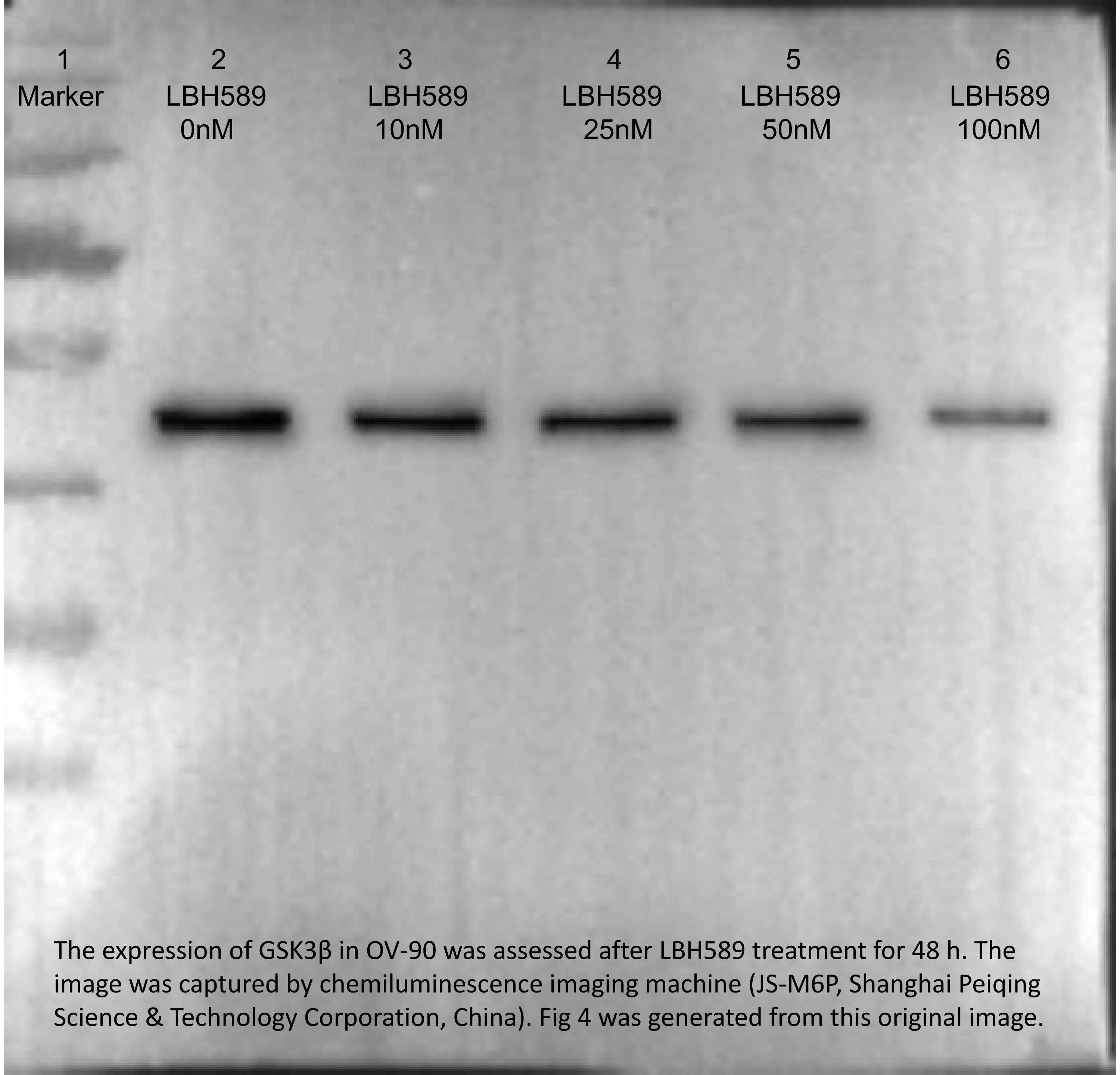

The expression of GSK3β in OV-90 was assessed after LBH589 treatment for 48 h. The image was captured by chemiluminescence imaging machine (JS-M6P, Shanghai Peiqing Science & Technology Corporation, China). Fig 4 was generated from this original image.

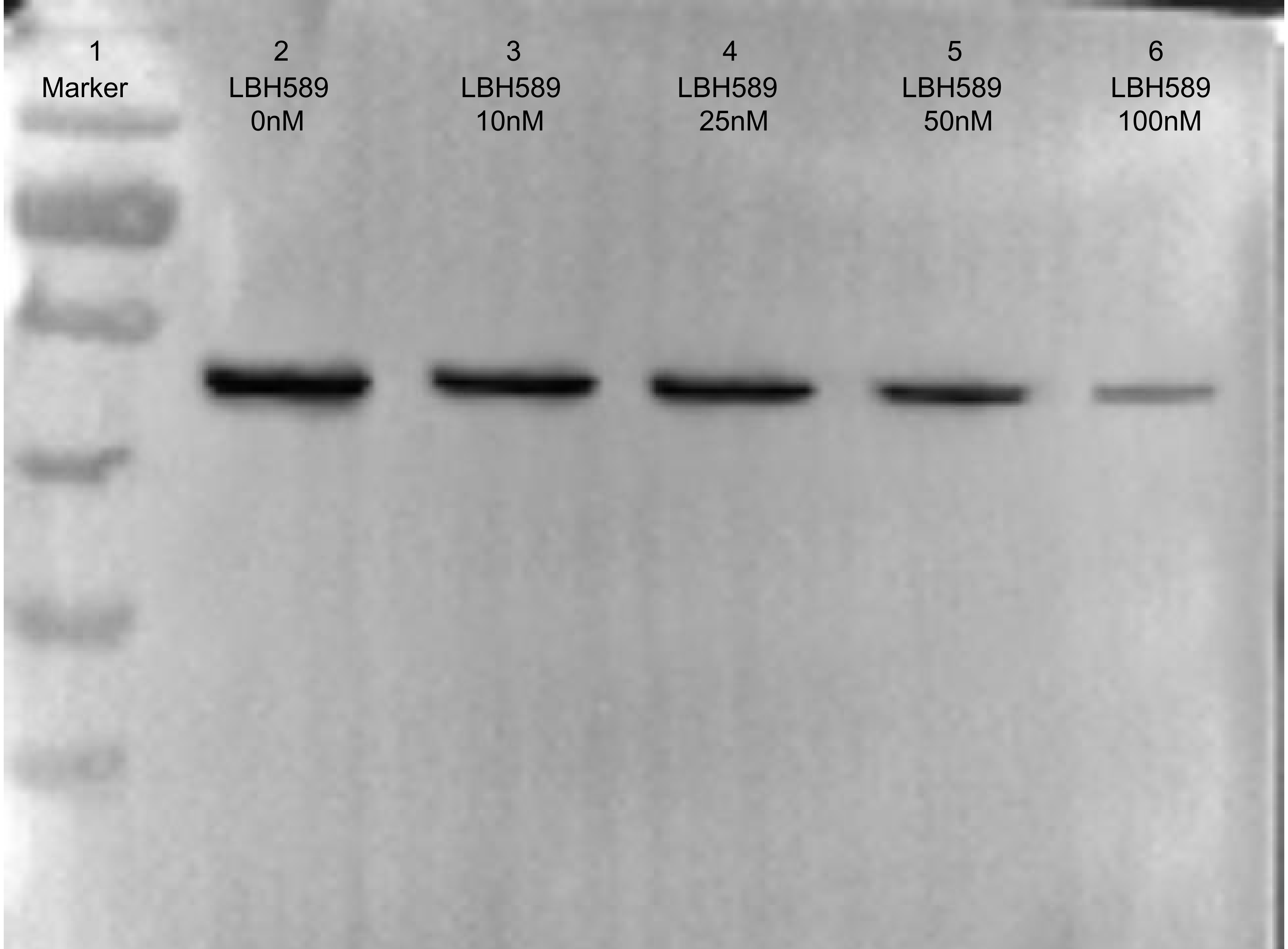

The expression of GSK3β in SKOV-3 was assessed after LBH589 treatment for 48 h. The image was captured by chemiluminescence imaging machine (JS-M6P, Shanghai Peiqing Science & Technology Corporation, China). Fig 4 was generated from this original image.

The expression of pVHL in OV-90 was assessed after LBH589 treatment for 48 h. The image was captured by chemiluminescence imaging machine (JS-M6P, Shanghai Peiqing Science & Technology Corporation, China). Fig 5 was generated from this original image.

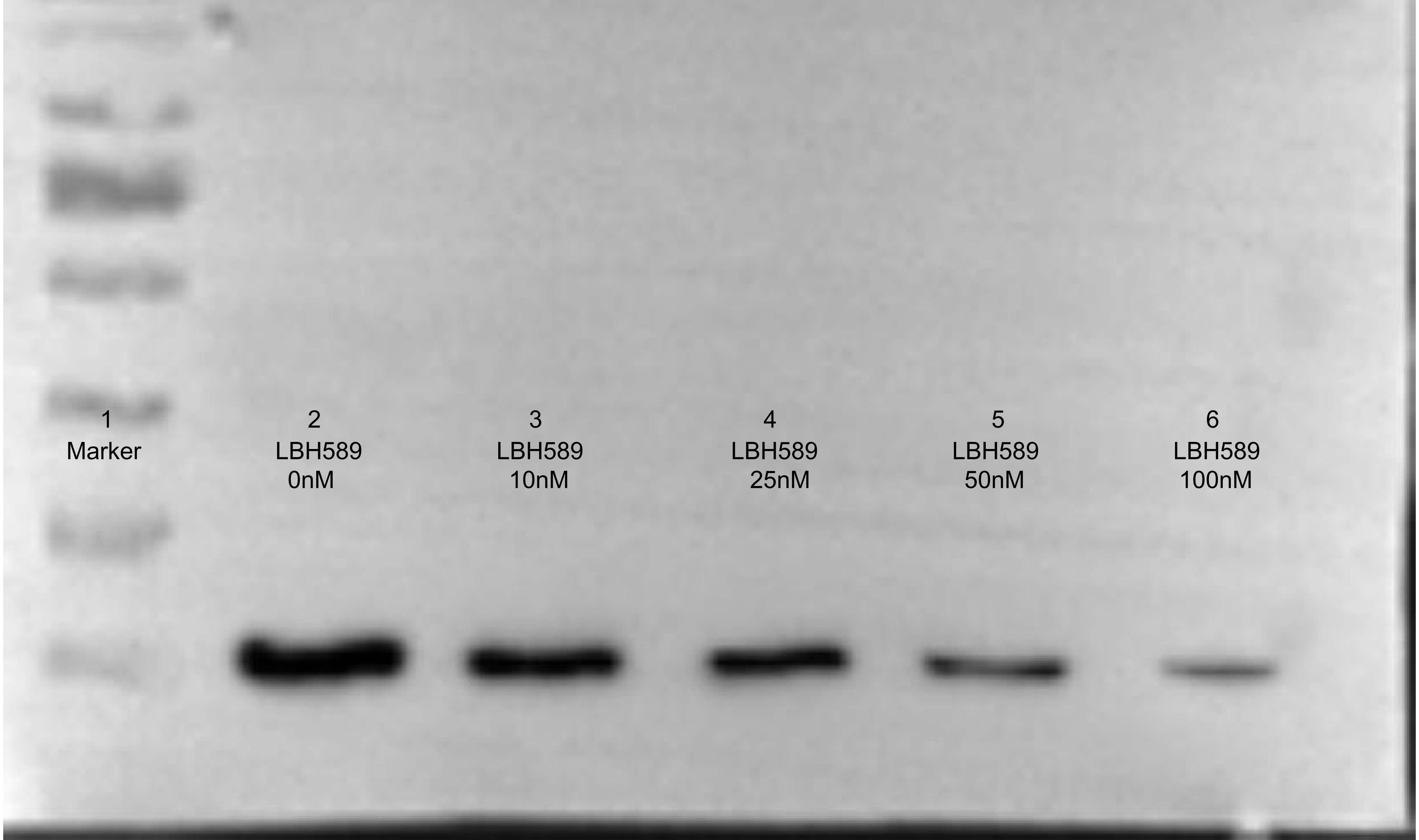

The expression of pVHL in SKOV-3 was assessed after LBH589 treatment for 48 h. The image was captured by chemiluminescence imaging machine (JS-M6P, Shanghai Peiqing Science & Technology Corporation, China). Fig 5 was generated from this original image.

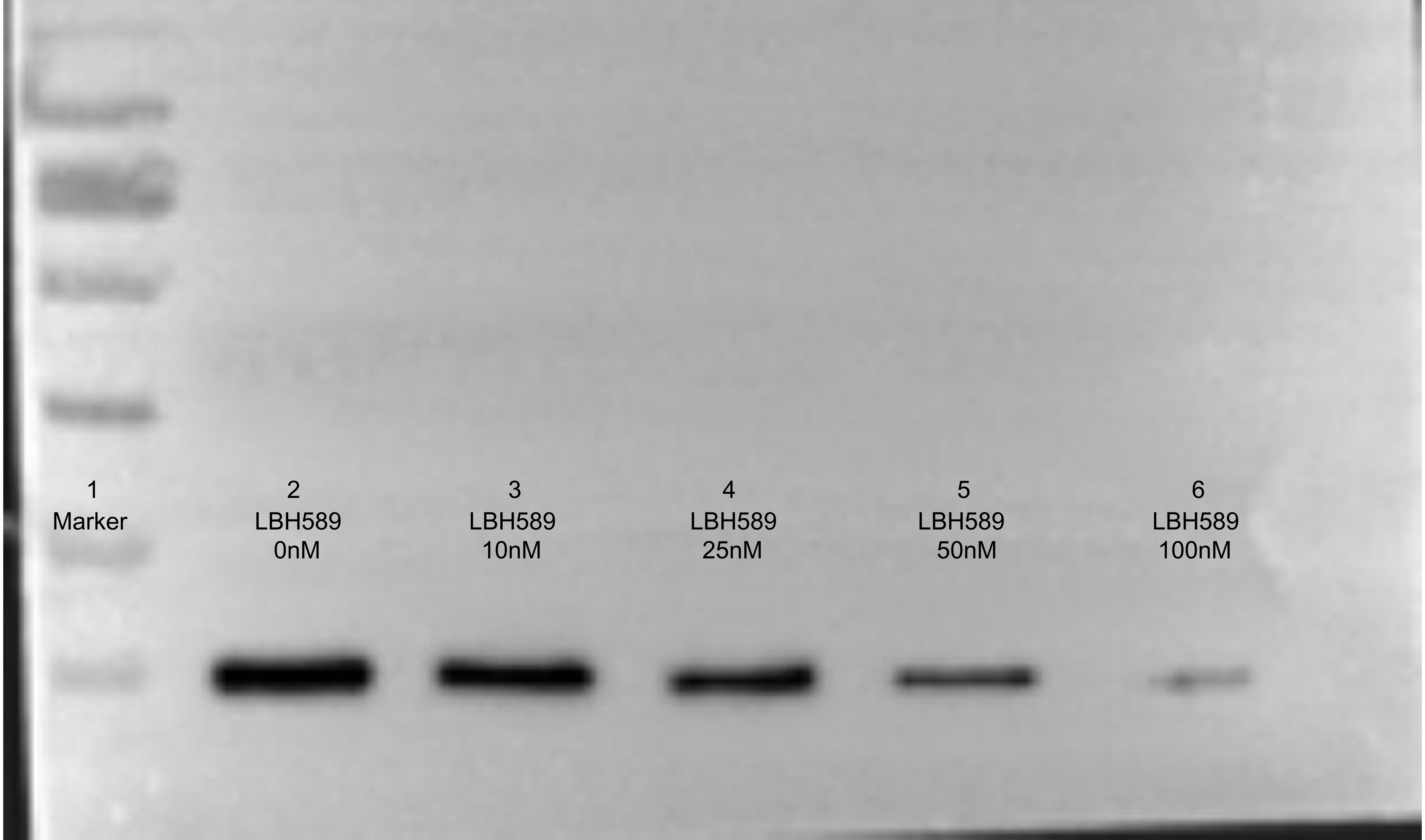

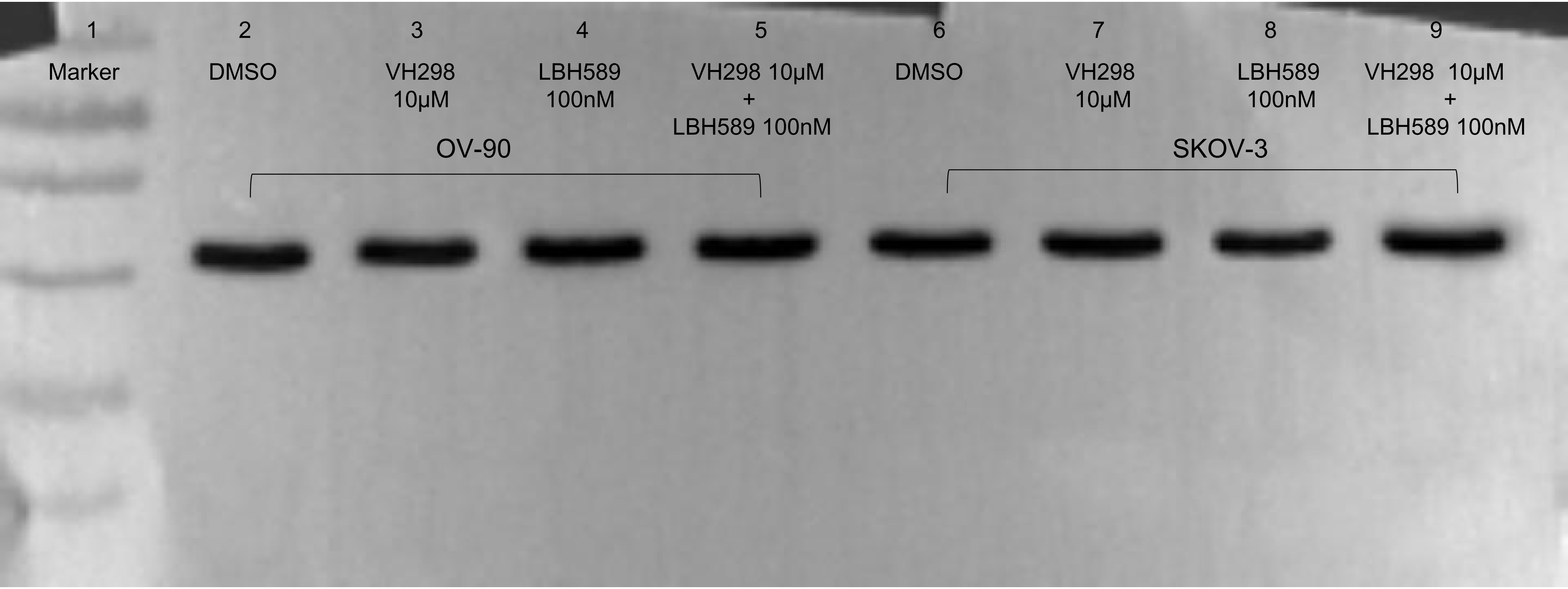

The expression of β actin was assessed after treatment of VH298 or LBH589 for 48 h. The image was captured by chemiluminescence imaging machine (JS-M6P, Shanghai Peiqing Science & Technology Corporation, China). Fig 6 was generated from this original image.

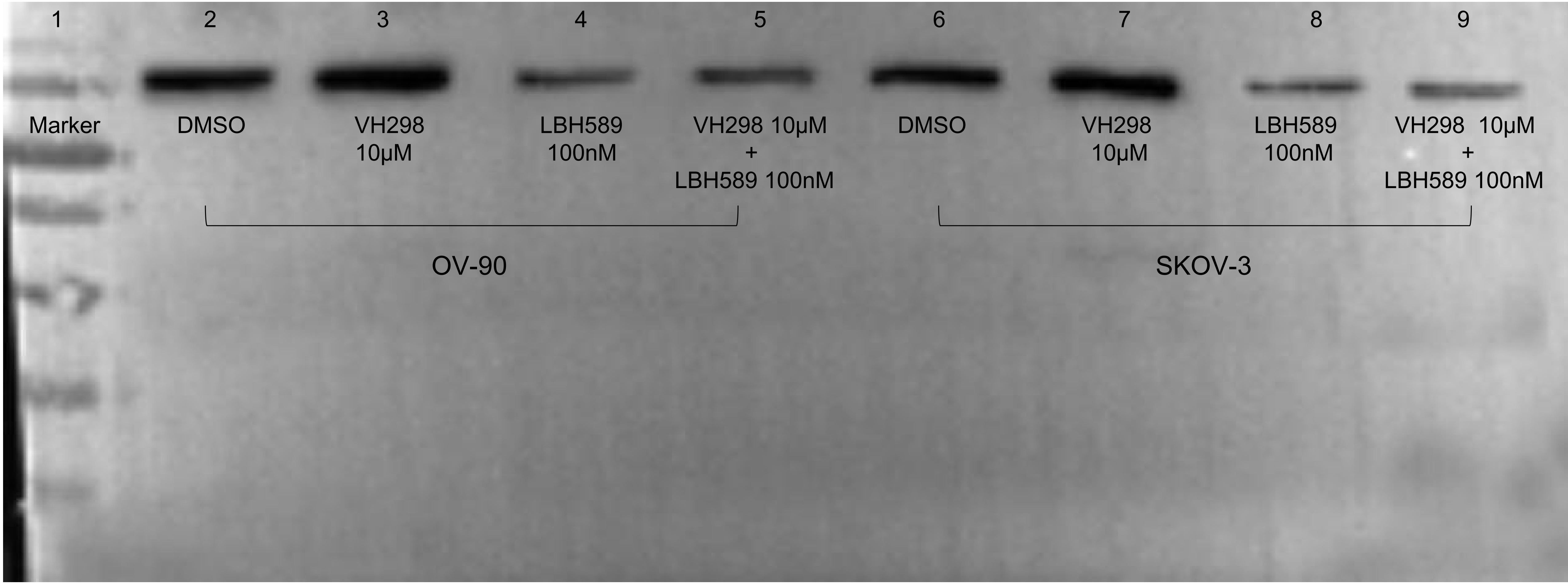

The expression of HIF-1α was assessed after treatment of VH298 or LBH589 for 48 h. The image was captured by chemiluminescence imaging machine (JS-M6P, Shanghai Peiqing Science & Technology Corporation, China). Fig 6 was generated from this original image.

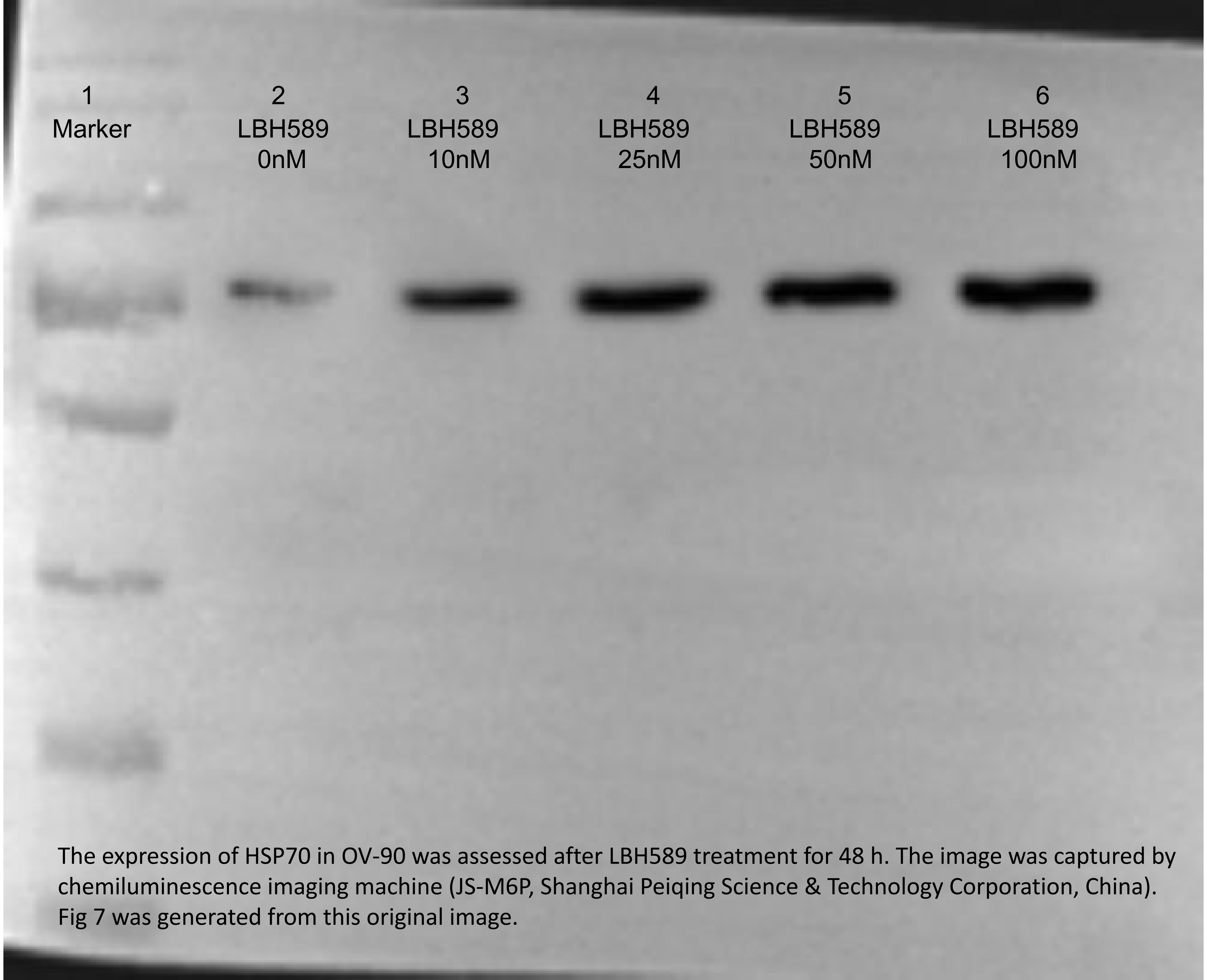

The expression of HSP70 in OV-90 was assessed after LBH589 treatment for 48 h. The image was captured by chemiluminescence imaging machine (JS-M6P, Shanghai Peiqing Science & Technology Corporation, China). Fig 7 was generated from this original image.

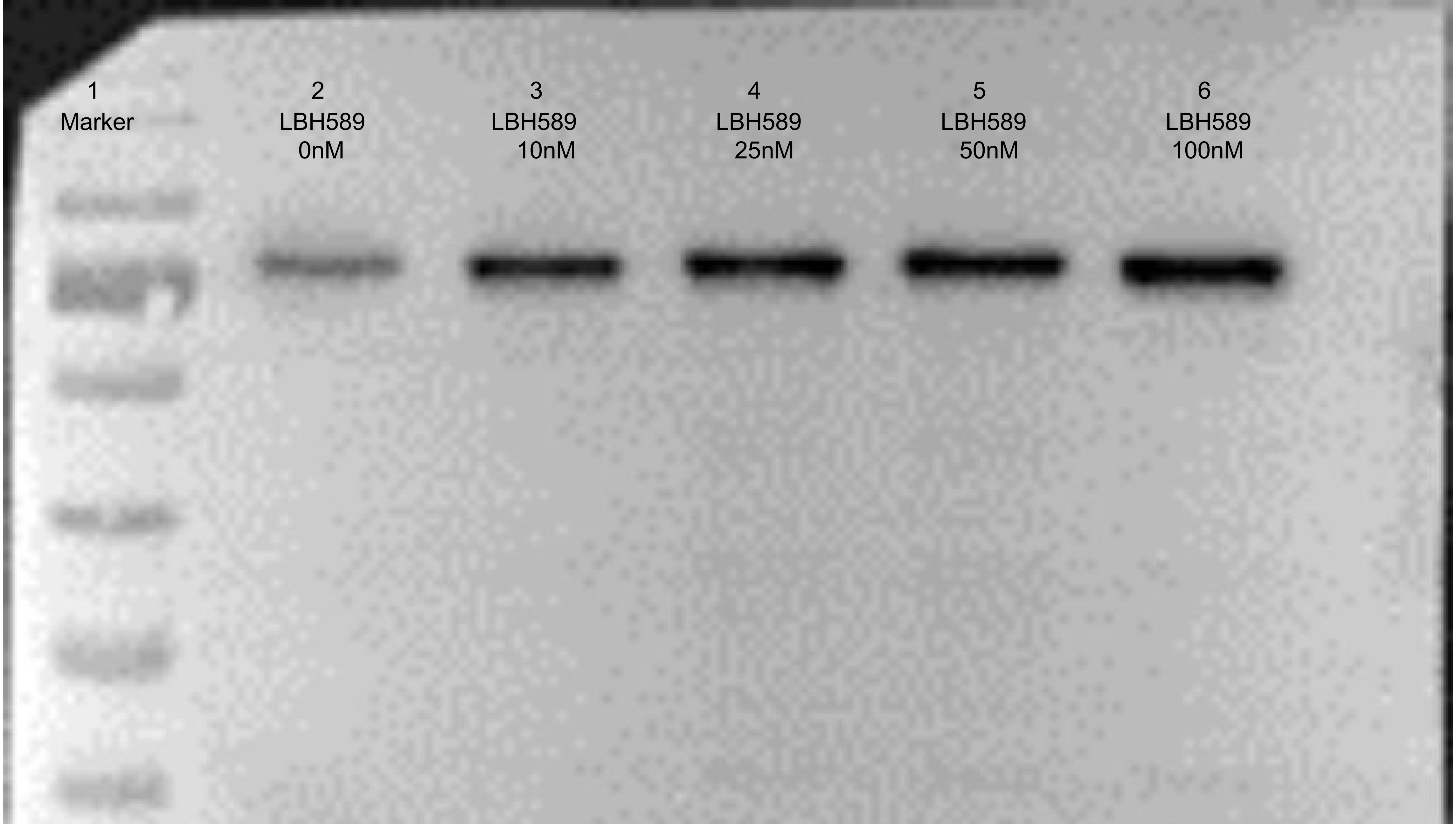

The expression of HSP70 in SKOV-3 was assessed after LBH589 treatment for 48 h. The image was captured by chemiluminescence imaging machine (JS-M6P, Shanghai Peiqing Science & Technology Corporation, China). Fig 7 was generated from this original image.

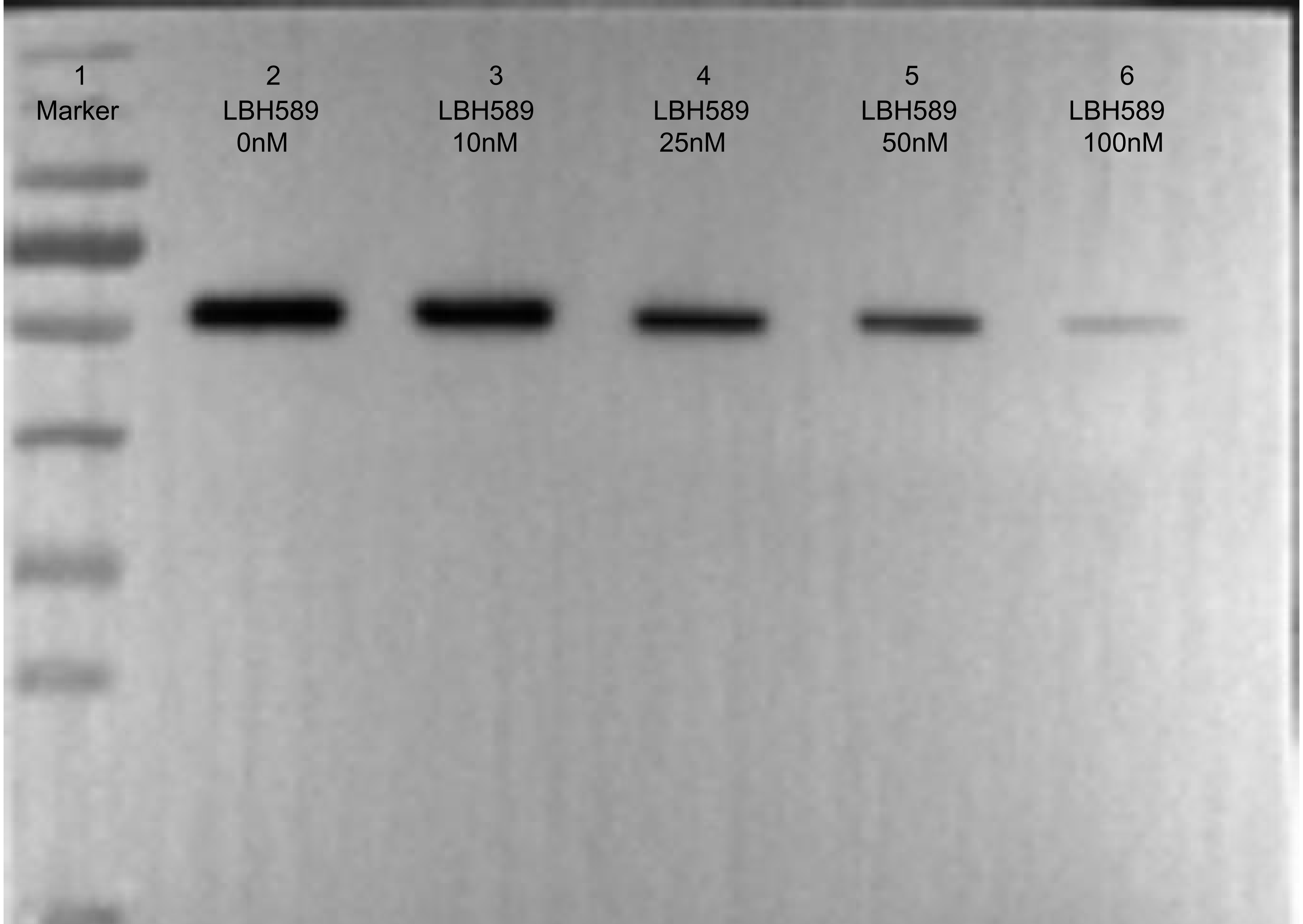

The expression of TCP-1α in OV-90 was assessed after LBH589 treatment for 48 h. The image was captured by chemiluminescence imaging machine (JS-M6P, Shanghai Peiqing Science & Technology Corporation, China). Fig 8 was generated from this original image.

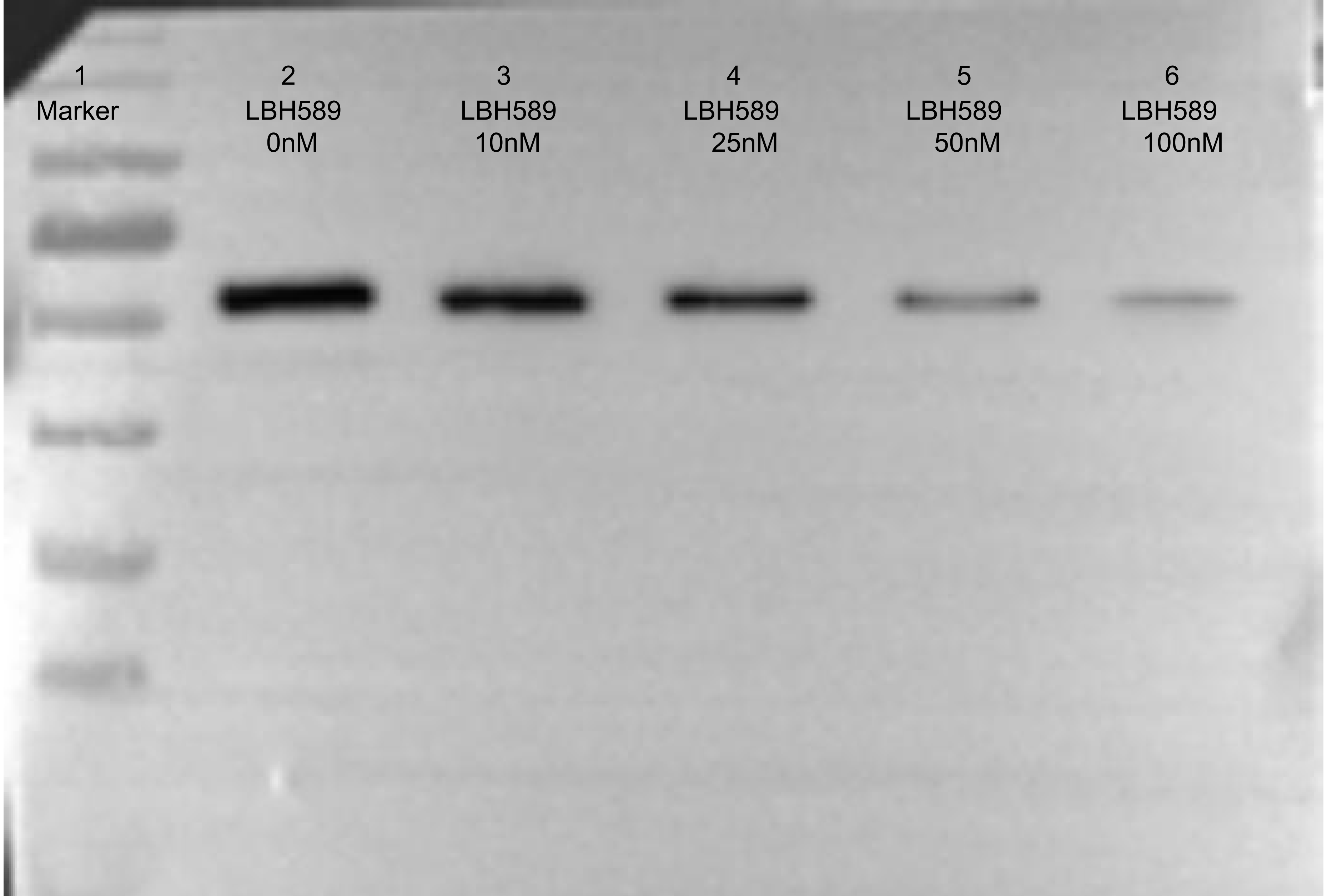

The expression of TCP-1α in SKOV-3 was assessed after LBH589 treatment for 48 h. The image was captured by chemiluminescence imaging machine (JS-M6P, Shanghai Peiqing Science & Technology Corporation, China). Fig 8 was generated from this original image.

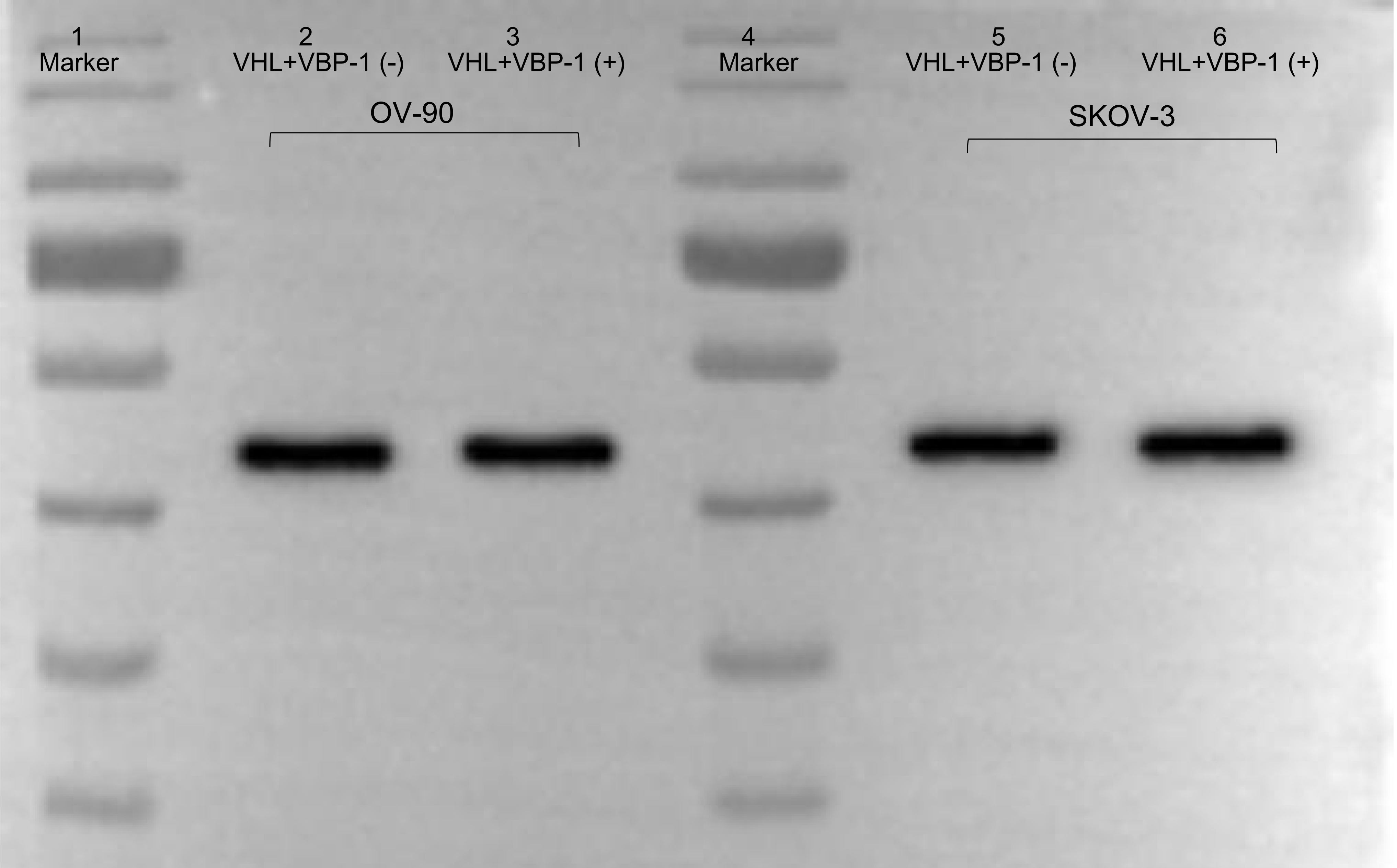

The expression of β actin was assessed after transfection of plasmids of VHL and VBP-1 for 48 h. The image was captured by chemiluminescence imaging machine (JS-M6P, Shanghai Peiqing Science & Technology Corporation, China). Fig 9, 10 were generated from this original image.

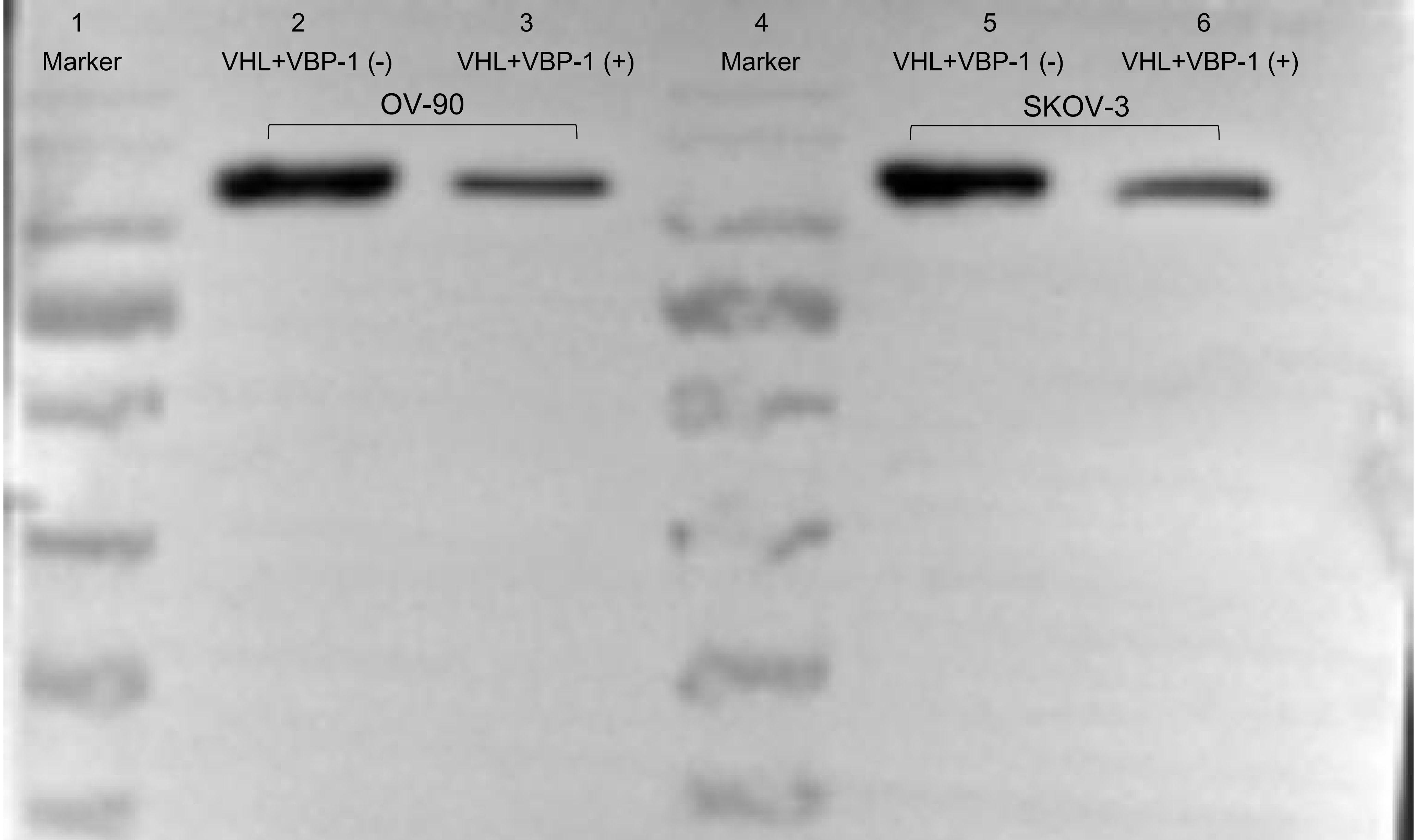

The expression of HIF-1α was assessed after transfection of plasmids of VHL and VBP-1 for 48 h. The image was captured by chemiluminescence imaging machine (JS-M6P, Shanghai Peiqing Science & Technology Corporation, China). Fig 9 was generated from this original image.

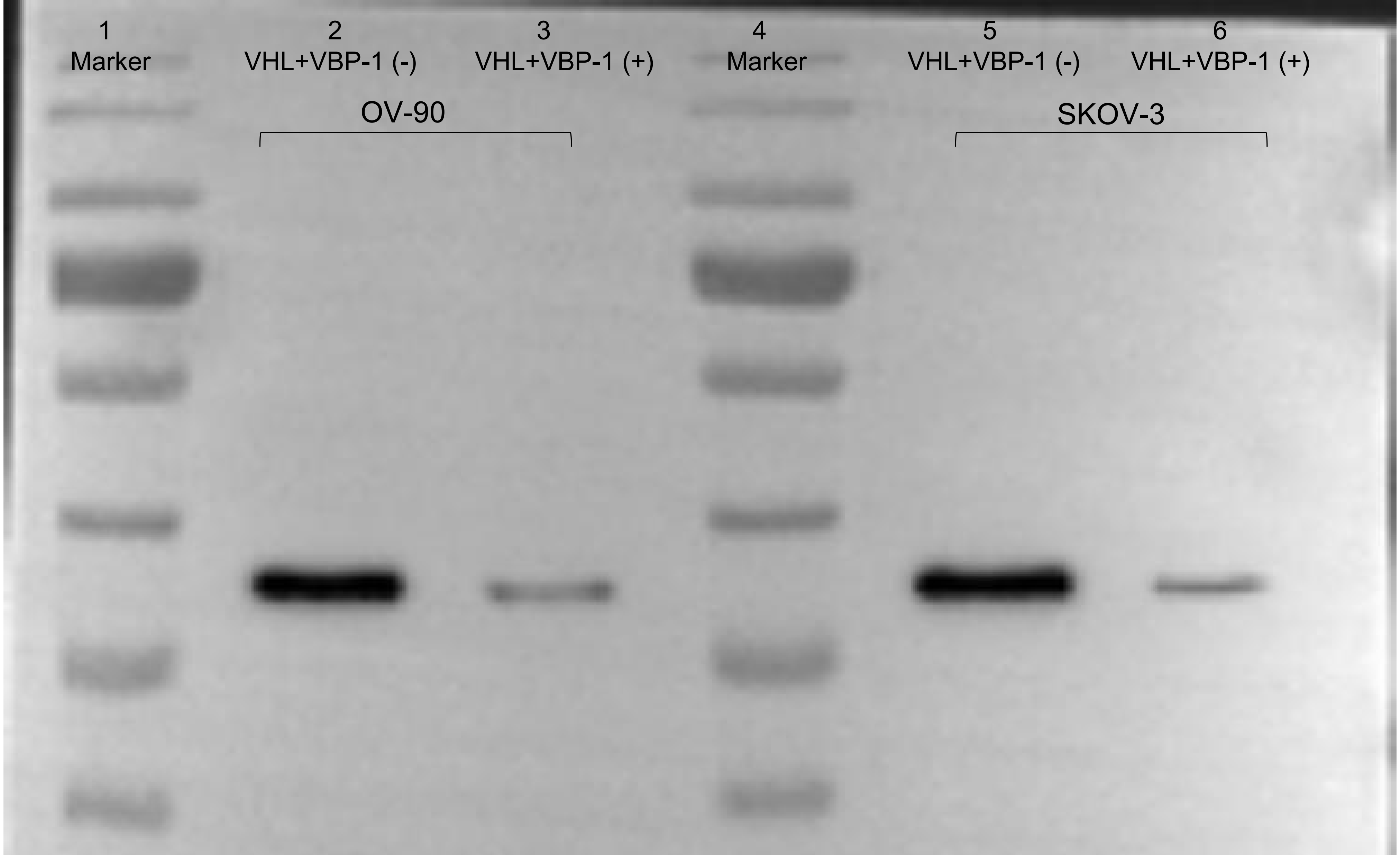

The expression of LDHA was assessed after transfection of plasmids of VHL and VBP-1 for 48 h. The image was captured by chemiluminescence imaging machine (JS-M6P, Shanghai Peiqing Science & Technology Corporation, China). Fig 10 was generated from this original image.
